# Body mass index and waist circumference trajectories among midlife women in the United States and their association with incident diabetes: The Study of Women’s Health Across the Nation

**DOI:** 10.1371/journal.pone.0357147

**Published:** 2026-11-12

**Authors:** Tanesha Brewster, Sharifa Nasreen

**Affiliations:** Department of Epidemiology and Biostatistics, School of Public Health, SUNY Downstate Health Sciences University, Brooklyn, New York, United States of America; Trinium Woman’s Hospital, KOREA, REPUBLIC OF

## Abstract

**Background:**

This study aimed to identify the trajectories of BMI and waist circumference (WC) in middle-aged women in the U.S. and examine their association with incident diabetes.

**Methods:**

We used secondary data from the longitudinal Study of Women’s Health Across the Nation over 10 annual visits (1996–2008). Latent class growth modeling was used to identify BMI and WC trajectory groups during visits 1–5. Cox proportional hazards regression models were used to estimate the hazard ratios (HRs) for diabetes during visits 6–10.

**Results:**

Among 1,406 women, 90 (6.4%) developed diabetes. Four BMI trajectory groups were identified: high increasing (19.5%), low stable (37.4%), medium increasing (34.8%), and very high increasing (8.3%) with HRs for diabetes of 3.83 (95% CI: 1.76, 8.31) in the medium increasing, 6.97 (95% CI: 3.00, 16.16) in high increasing, and 14.22 (5.92, 34.17) in very high increasing trajectory groups. Four WC trajectory groups were identified: increasing (35.9%), normal increasing (38.4%), high increasing (18.7%), and very high stable (6.9%); HRs for diabetes were 2.26 (95% CI: 1.06, 4.85) in increasing, 8.12 (95% CI: 3.64, 18.11) in high increasing, and 11.30 (95% CI: 4.69, 27.25) in very high stable groups. Four multi-trajectories of BMI and WC were identified: group 1 (34.5%, high increasing BMI and increasing WC), group 2 (33.1%, low stable BMI and normal increasing WC), group 3 (22.1%, medium increasing BMI and high increasing WC), and group 4 (10.3%, very high increasing BMI and very high increasing WC) with a higher rates of diabetes in groups 1, 3, and 4.

**Conclusions:**

Middle-aged women followed distinct BMI and WC trajectory groups that were associated with incident diabetes. Public health interventions on weight management could help reduce the burden of diabetes in this population.

## Background

In 2021, globally, 529 million people had diabetes with a global prevalence of 6.1% [1], and diabetes was directly accounted for 1.6 million deaths [2]. With the increasing global prevalence, more than 1.31 billion people are estimated to have diabetes by 2050 [1]. In the United States (US), 38.4 million people of all ages had diabetes in 2021 affecting 11.6% of the total population; there were 1.2 million new cases of diabetes in 2021 with an incidence rate of 5.9 per 1,000 people [3].

Menopause is the biological transition that naturally occurs in every woman’s life at the end of reproductive years. Natural menopause is clinically defined retrospectively after 12 consecutive months of cessation of menstruation due to decreased ovarian function in absence of any other physiological or pathological cause, or clinical intervention [4]. This transition to decreased endogenous oestradiol is associated with an array of phenotypical, metabolic, and biochemical changes such as increase in and central redistribution of adipose tissue leading to central and visceral adiposity, decrease in energy expenditure, impairment of insulin secretion and insulin sensitivity that increases the risk of type 2 diabetes [5]. An increased risk of diabetes has been observed with overweight and obesity in premenopausal and postmenopausal women [6–8].

BMI can serve as an important indicator for assessing obesity-related health risks. However, as BMI changes over time, the traditional way of examining the baseline BMI or a change in the BMI is unlikely to capture the dynamic nature of BMI and its association to the development of different health outcomes, including type 2 diabetes. Few studies have examined the trajectories of BMI among menopausal women and their association with the risk of diabetes. One study found differential risk of developing diabetes mellitus with seven different BMI trajectories, particularly an increased risk with a rapid weight increase in Australian women [9].

While BMI is the most commonly used indicator to measure obesity, BMI is limited in its ability to distinguish between fat mass and non-fat lean mass and to capture the distribution of fat over the body [10]. Abdominal fat mass, also known as abdominal, central or visceral obesity, results from an accumulation of abdominal fat, which is a risk factor for metabolic diseases including diabetes, and overall mortality [11]. It has been suggested that BMI alone is inadequate to evaluate the risks of cardiometabolic disorders related to adult adiposity [12]. An increased odds of diabetes was found among African American women [13]. Waist circumference trajectory was also found to be a predictor of diabetes in middle-aged Korean population [14]. Another study in European women found that the association between waist circumference and the risk of developing diabetes varied by the BMI categories, suggesting that the use of BMI or waist circumference alone would be insufficient to adequately assess the risk of diabetes [15].

There are limited studies examining the trajectories of BMI and waist circumference and their association with incident diabetes. Further studies are needed to identify groups of middle aged women who follow similar trajectory groups of these two parameters to capture their interrelationship and examine the association with the risk of developing diabetes. As BMI and waist circumference are modifiable risk factors, identifying risk groups will be useful in developing targeted prevention strategies. The objectives of this study were to identify the trajectories of BMI and waist circumference in middle aged women in the United States, and to examine the association between the trajectory groups and incident diabetes mellitus.

## Materials and methods

### Study population

This study used de-identified data from the Study of Women’s Health Across the Nation (SWAN)*,* a multi-site, multi-ethnic longitudinal study among 3,302 midlife women aged 42–52 years at recruitment during 1996–1997 from seven sites (Boston, Chicago, the Detroit area, Los Angeles, Newark, Pittsburgh, and Oakland California) across the US to characterize the physical, biological, psychological, and social changes during menopausal transition [16]. This current study used publicly available data for women followed annually over a period of 10 years from 1996–1997 through 2006–2008 [17–28]. The data were accessed in January 2025 for this research. This study used publicly available de-identified secondary data from the SWAN study accessed through the Inter-university Consortium for Political and Social Research (ICPSR). The original study was approved by the Institutional Review Boards at each participating site, and informed consent was obtained from all participants. Because this study used publicly available de-identified data, it is deemed not to constitute human subjects research. As such, ethical IRB review and approval was not needed.

For this study, pre- and early perimenopausal women without diabetes at baseline were included. Premenopausal status was defined as having menstruation in the previous three months with no change in menstrual regularity in the past year, and early perimenopausal status was defined as having menstruation in the previous three months with changes in regularity in the past year. Women with missing diabetes status or those who developed diabetes during the BMI or waist circumference measurement period (first to fifth visits), women with fewer than three BMI or waist circumference measurements during first to fifth visits (women with one or two missing measurements were included), women who experienced pregnancy during the study period, and women with missing covariates at baseline were excluded (Fig 1). The study period was divided into two time periods: a 5-year period (first to fifth visits) to identify the BMI and waist circumference trajectory groups (the BMI or waist circumference measurement period), and a subsequent 5-year period (sixth to tenth visits) to identify incident diabetes.

**Fig 1:**
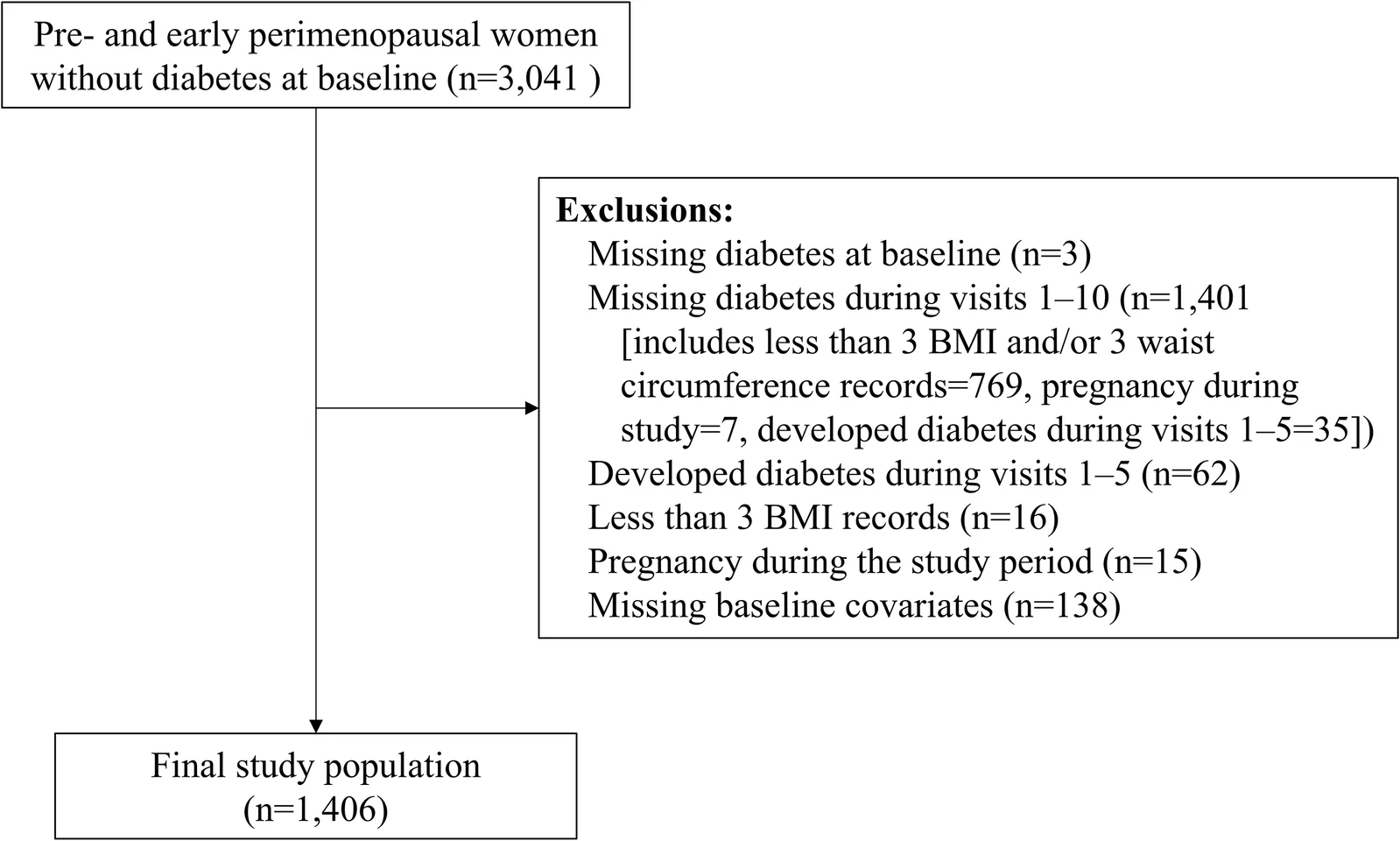
Participant selection.

### Diabetes, BMI, waist circumference and covariates

Incident diabetes was defined from self-report of healthcare provider diagnosis or treatment of diabetes ascertained from an affirmative answer to the question “Since your last study visit, has a doctor, nurse practitioner or other health care provider told you that you had any of the following conditions or treated you for them - diabetes?” asked at each visit. The BMI was calculated as weight in kilograms divided by the square of height in meters using height (measured using stadiometer or portable machine) and weight (measured using a balance beam, clinic digital, or portable scale) measured at each visit. We categorized BMI according to the standard cutoffs: underweight (<18.5 kg/m^2^), healthy weight (18.5–24.9 kg/m^2^), overweight (25–29.9 kg/m^2^), and obesity (≥30 kg/m^2^); obesity was further categorized as class 1 obesity (30–34.9 kg/m^2^), class 2 obesity (35–39.9 kg/m^2^), and class 3 obesity (40 or greater kg/m^2^) [29]. Weight circumference (cm) was measured at each visit. We categorized waist circumference for risk of metabolic complications according to the World Health Organization’s standard for female: normal risk (waist circumference <80 cm), increased risk (waist circumference 80–88 cm), and substantially increased risk (waist circumference >88 cm) [11]. The following covariates collected at baseline and self-reported were included: age in years, race/ethnicity (Black/African American, Chinese/Chinese American, Japanese/Japanese American, Caucasian/White Non-Hispanic, and Hispanic), educational level (high school graduate, some college/technical school, college graduate, post graduate education), total family income ($20,000 to $49,999, $50,000 to $99,000, $100,000 or more), smoking status (non-smoker/smoker), played sports/exercised during the past year as a proxy of physical activity (never, less than once a month, once a month, 2–3 times a month, once a week, more than once a week), and family history (in parents or siblings) of diabetes (no/yes).

### Statistical analysis

Baseline characteristics of the study participants were summarized as mean (standard deviation, SD), and median (interquartile range [IQR]) for continuous variables, and as frequency (percentage) for categorical variables. We conducted Spearman’s correlation to assess the relationship between BMI and waist circumference.

We used the latent class growth modeling, a semi-parametric group-based trajectory modeling, to identify distinct groups of women with similar patterns of BMI trajectory over time [30]. First, we identified the number of latent trajectory groups by testing models with up to 5 trajectory groups (15 candidate models). The censored normal distribution was used to model BMI trajectories, and the shape of the trajectory was defined by a polynomial function of age in years at each visit. Each model initially included linear, quadratic, and cubic polynomial terms for age at each visit during the measurement period to allow the possibility of nonlinear relationship between BMI and age. Models with different numbers of trajectory groups were compared using the Bayesian Information Criterion (BIC) as a measure of goodness-of-fit, with the requirement that each group included at least 5% of the study participants. BIC values for the competing models are presented in S1 Table. The four-group model was selected based on the combined consideration of BIC, minimum group size, trajectory separation, and clinical interpretability. Compared with the three-group model, the four-group model distinguished two clearly separated intermediate BMI trajectories that were combined into a single trajectory in the three-group model. The four trajectories also yielded four clinically distinguishable BMI trajectories corresponding approximately to healthy weight, overweight, class 1 obesity and class 3 obesity with all groups exceeding the 5% minimum group size. The five-group model included an additional trajectory group consisting of only 2.1% of participants below the prespecified 5% minimum group size. Then the results of statistical significance testing of the 3 polynomial terms for age were checked to see whether the quadratic and cubic terms were statistically significant or not. The quadratic and cubic terms were excluded if they were not statistically significant; linear terms were included in the model regardless of statistical significance. The final model included four groups with a cubic polynomial term for the first group, and linear terms for the remaining groups. Finally, we assessed the adequacy of the final model by 3 diagnostics: average posterior probability of each group more than 0.70, odds of correct classification more than 5, and the model estimated group membership probability differed by less than 50% from the proportion assigned to a group based on the largest posterior probability [31]. The baseline characteristics were then summarized according to the identified BMI trajectory groups and compared using a one-way analysis of variance (ANOVA) for the continuous variable (age), and χ2 (chi-square) test for the categorical variables. We estimated the rates of diabetes per 1000 person-years for each trajectory group. Then, we used Cox proportional hazards regression models (unadjusted and adjusted) to examine the association between BMI trajectory groups and incident diabetes by estimating hazard ratios (HRs) and 95% confidence intervals (CIs). The following variables were adjusted for in the adjusted analysis: menopausal status, race/ethnicity, education, total family income, smoking status, played sports/exercised during the past year, and family history of diabetes. These covariates were selected a priori based on their clinical relevance to adiposity and diabetes risk, and their potential to account for baseline differences between adiposity trajectory groups. Baseline menopausal status was included to account for differences in adiposity patterns and diabetes risk associated with menopausal status. We used Schoenfeld global test to test for the proportional-hazards assumption.

We used a similar method to identify waist circumference trajectory groups. The final model included four groups, all with linear terms. We summarized and compared the baseline characteristics according to the identified waist circumference trajectory groups using ANOVA and χ2 (chi-square) tests as appropriate. We estimated the rates of diabetes, and used Cox proportional hazards regression models to examine the association between waist circumference trajectory groups and incident diabetes by estimating the HRs and 95% CIs.

Finally, we conducted group-based multi-trajectory modeling to identify latent clusters (groups) of women following similar trajectories of BMI and waist circumference to capture interrelationship between the two [32]. We estimated the rates of diabetes and used Cox proportional hazards regression models to examine the association between the groups and incident diabetes by estimating the HRs and 95% CIs.

All analyses were performed using STATA 18 software (Stata Corp LP, College Station TX), and statistical-significance tests were carried out at the alpha level of 0.05 (2-sided). Latent class growth modelling was conducted using the “*traj*” procedure in STATA [33].

## Results

A total of 1,406 women were included in the analysis (Fig 1). Among them, 56% were pre-menopausal, and 44% early perimenopausal at baseline (Table 1). At baseline, the mean age (SD) was 45.9 (2.7) years. Most of the participants were Caucasian (54%), had some college or technical degree (31%), had total family income of $50,000 to $99,000 (42%), were not current smoker (89%), played sports or exercised more than once a week in the past year (40%), and did not have any family history of diabetes (63%) (Table 1). There was a statistically significant strong positive correlation between BMI and waist circumference (Spearman correlation coefficient, *r*_*s*_ = 0.9289, p=<0.0001).

**Table 1:** Baseline characteristics of women.

Characteristics	Frequency (%) (N = 1,406)
Menopause Status	
Pre-menopausal	785 (55.8)
Early perimenopause	621 (44.2)
Age in years	
Mean (SD)	45.9 (2.7)
Median (IQR)	46.0 (44, 48)
Race/ethnicity	
Black/African American	330 (23.5)
Chinese/Chinese American	146 (10.4)
Japanese/Japanese American	177 (12.6)
Caucasian/White Non-Hispanic	753 (53.6)
Educational level	
Less than High School	35 (2.5)
High school graduate	188 (13.4)
Some college/technical school	436 (31.0)
College graduate	341 (24.3)
Post graduate education	406 (28.9)
Total family income	
Less than $19,999	93 (6.6)
$20,000–49,999	451 (32.1)
$50,000–99,999	587 (41.8)
$100,000 or more	275 (19.6)
Smoking status	
Not a current smoker	1,252 (89.1)
Current smoker	154 (10.9)
Played sports/ exercised during past year	
Never	254 (18.1)
Less than once a month	182 (12.9)
Once a month	71 (5.1)
2–3 times a month	167 (11.9)
Once a week	164 (11.7)
More than once a week	568 (40.4)
Family History of diabetes mellitus	
No	884 (62.9)
Yes	469 (33.4)
Unknown	53 (3.8)

SD, standard deviation; IQR, interquartile range.

### Trajectory groups of BMI

BMI trajectory during the first five visits was best fitted by a four-group model (Fig 2). The estimates of the trajectory parameters and model adequacy information are presented in S2 Table. The first trajectory group (*high increasing*) comprised of 19.5% women with class 1 obesity, and BMI increased gradually but remained within the class 1 obesity. The second trajectory group (*low stable*) comprised of 37.4% women with a healthy weight, and BMI increased slightly but remained stable within healthy weight range during the BMI measurement period. The third trajectory group (*medium increasing*) comprised of 34.8% women who were overweight, and BMI gradually increased during the BMI measurement period but remained within the overweight category. The fourth trajectory group (*very high increasing*) comprised of 8.3% women with class 3 obesity where BMI increased slightly during the BMI measurement period.

**Fig 2:**
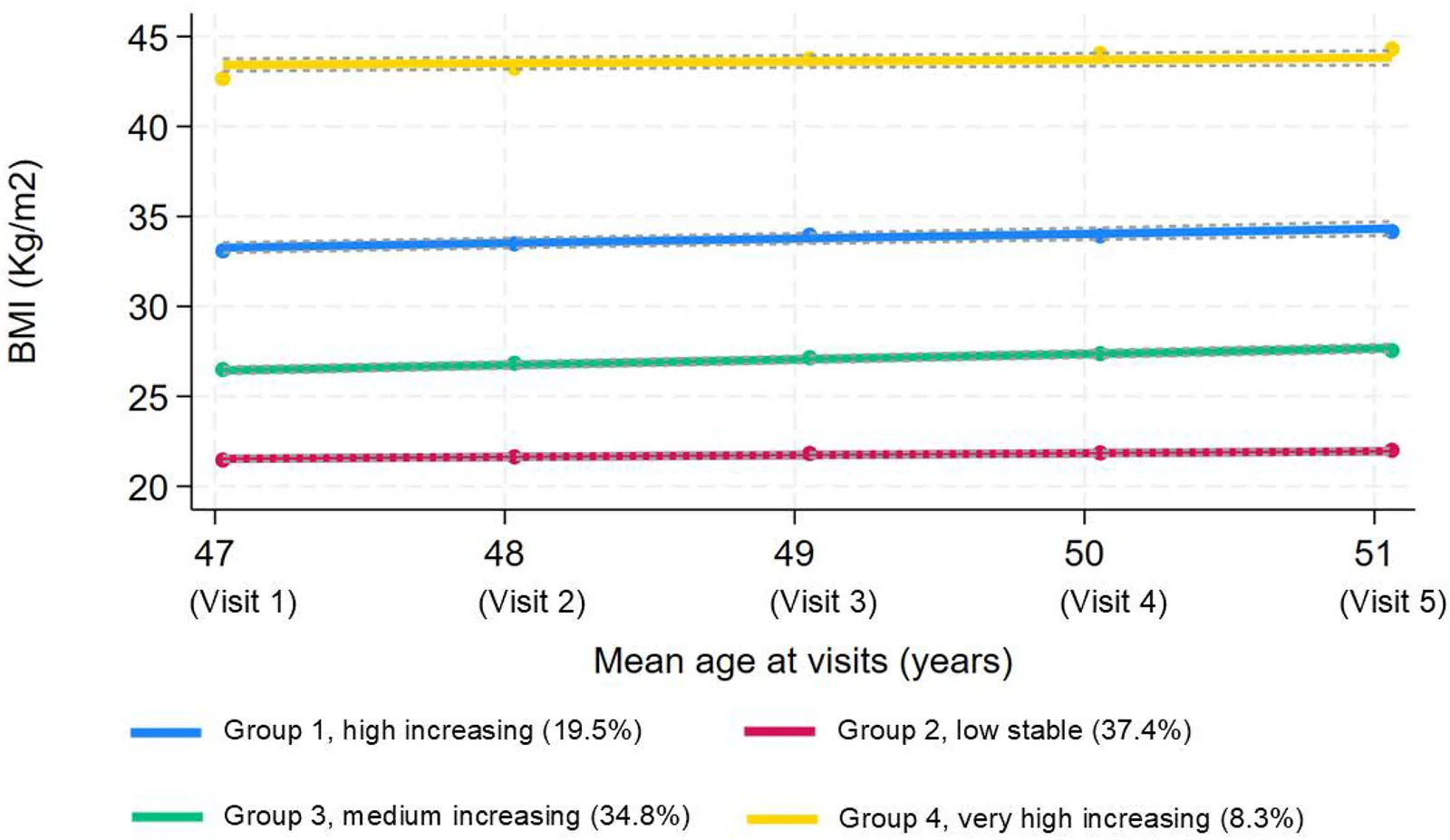
Body mass index (BMI) trajectory groups during the measurement period. Solid lines represent the estimated trajectories, dot symbols represent the observed group means at each age, and the dashed lines represent 95% pointwise confidence intervals on the estimated trajectories.

### Baseline characteristics according to BMI trajectory groups

The baseline characteristics of the women according to the BMI trajectory groups are presented in Table 2. The age of women in the trajectory groups were similar. There were statistically significant differences in menopausal status, race/ethnicity, educational level, total family income, played sports/exercise in past year and family history of diabetes among the trajectory groups. More than half of the women in trajectory group 1 were in early perimenopause, while more than half of the women in trajectory groups 2, 3, and 4 were premenopausal at baseline. Most women in trajectory groups 1, 3, and 4 were Caucasian/White Non-Hispanic or Black/African American. Trajectory group 2 had a lower proportion of Black/African American but a higher proportion of Chinese/Chinese American and Japanese/Japanese American than other trajectory groups. The highest proportion of women in trajectory group 2 had post graduate education, whereas the highest proportion of women in other trajectory groups had some college/technical school level education. The highest proportion of women in trajectory groups 1, 2, and 3 had a total family income of $$50,000 to $99,999, whereas the highest proportion of women in trajectory group 4 had a total family income of 20,000 to $49,999. A higher proportion of women in trajectory group 4 reported that they never played sports or exercised in the past year compared to women in trajectory groups 1, 2, and 3. A higher proportion of women in trajectory group 4 had a family history of diabetes mellitus than women in the other trajectory groups.

**Table 2:** Baseline characteristics according to body mass index (BMI) trajectory groups.

Characteristics	Frequency (%)				P value
	Trajectory group 1, high increasing (n = 271)	Trajectory group 2, low stable (n = 524)	Trajectory group 3, medium increasing (n = 494)	Trajectory group 4, very high increasing (n = 117)	
Menopausal status					0.007
Pre-menopausal	124 (45.76)	301 (57.44)	291 (58.91)	69 (58.97)	
Early perimenopause	147 (54.24)	223 (42.56)	203 (41.09)	48 (41.03)	
Age in years					
Mean (SD)	45.54 (2.60)	46.03 (2.58)	46.02 (2.73)	45.78 (2.76)	0.555
Median (IQR)	45 (43, 47)	46 (44, 48)	46 (44, 48)	46 (43, 48)	
Race/ethnicity					<0.001
Black/African American	117 (43.17)	44 (8.40)	108 (21.86)	61 (52.14)	
Chinese/Chinese American	6 (2.21)	104 (19.85)	35 (7.09)	1 (0.85)	
Japanese/Japanese American	7 (2.58)	123 (23.47)	47 (9.51)	0 (0.00)	
Caucasian/White Non-Hispanic	141 (52.03)	253 (48.28) `	304 (61.54)	55 (47.01)	
Educational level					<0.001
Less than High	5 (1.85)	17 (3.24)	8 (1.62)	5 (4.27)	
High school graduate	53 (19.56)	60 (11.45)	51 (10.32)	24 (20.51)	
Some college/technical school	107 (39.48)	135 (25.76)	153 (30.97)	41 (35.04)	
College graduate	47 (17.34)	155 (29.58)	115 (23.28)	24 (20.51)	
Post graduate education	59 (21.77)	157 (29.96)	167 (33.81)	23 (19.66)	
Total family income					<0.001
Less than $19,999	26 (9.59)	21 (4.01)	23 (4.66)	23 (19.66)	
$20,000–49,999	106 (39.11)	147 (28.05)	149 (30.16)	49 (41.88)	
$50,000–99,999	107 (39.48)	221 (42.18)	218 (44.13)	41 (35.04)	
$100,000 or more	32 (11.81)	135 (25.76)	104 (21.05)	4 (3.42)	
Smoking status					0.233
Not a current smoker	236 (87. 08)	476 (90.84)	436 (88.26)	104 (88.89)	
Current smoker	35 (12.92)	48 (9.16)	58 (11.74)	13 (11.11)	
Played sports/exercised during past year					<0.001
Never	58 (21.40)	77 (14.69)	77 (15.59)	42 (35.90)	
Less than once a month	46 (16.97)	56 (10.69)	54 (10.93)	26 (22.22)	
Once a month	11 (4.06)	25 (4.77)	29 (5.87)	6 (5.13)	
2–3 times a month	44 (16.24)	44 (8.4)	65 (13.16)	14 (11.97)	
Once a week	36 (13.28)	62 (11.83)	57 (11.54)	9 (7.69)	
More than once a week	76 (28.04)	260 (49.62)	212 (42.91)	20 (17.09)	
Family History of diabetes mellitus					0.001
No	166 (61.25)	355 (67.75)	308 (62.35)	55 (47.01)	
Yes	103 (38.01)	149 (28.44)	164 (33.20)	53 (45.30)	
Unknown	2 (0.74)	20 (3.82)	22 (4.45)	9 (7.69)	

SD, standard deviation; IQR, interquartile range.

### Association between BMI trajectory groups and incident diabetes mellitus

Of the 1,406 women, 90 (6.4%) developed diabetes during visits 6–10. Among these incident diabetes, 10 developed in the low stable trajectory group (3.84 cases per 1000-person years), 25 in the medium increasing trajectory group (10.30 cases per 1000 person-years), 28 developed in the high increasing trajectory group (21.82 cases per 1000 person-years), and 27 in the very high increasing trajectory group (51.33 cases per 1000 person-years) (Table 3). The adjusted HRs for diabetes compared to the low stable group were 3.83 (95% CI: 1.76, 8.31) in medium increasing, 6.97 (95% CI: 3.00, 16.16) in high increasing, and 14.22 (5.92, 34.17) in very high increasing trajectory groups (Table 4).

**Table 3:** Incident diabetes and rates by trajectory groups.

Trajectory groups	Person-years	Incident diabetes, n (%) (N = 90)	Rate per 1000 person-years (95% CI)
**BMI trajectory groups**			
Low stable	2603	10 (11.1)	3.84 (2.07, 7.14)
Medium increasing	2427	25 (27.8)	10.30 (6.96, 15.24)
High increasing	1283	28 (31.1)	21.82 (15.07, 31.61)
Very high increasing	526	27 (30.0)	51.33 (35.20, 74.85)
**Waist circumference trajectory groups**			
Normal increasing	2661	12 (13.3)	4.51 (2.56, 7.94)
Increasing	2526	20 (22.2)	7.92 (5.12, 12.27)
High increasing	1220	35 (38.9)	28.69 (20.60, 39.96)
Very high stable	432	23 (25.6)	53.24 (35.38, 80.12)
**Multi-trajectory groups**			
Group 1	2416	21 (23.3)	8.69 (5.67, 13.33)
Group 2	2289	9 (10.0)	3.93 (2.05, 7.56)
Group 3	1481	29 (32.2)	19.58 (13.61, 28.18)
Group 4	653	31 (34.4)	47.47 (33.39, 67.50)

**Table 4:** Association between body mass index (BMI) trajectory groups and incident diabetes.

Trajectory group	Unadjusted		Adjusted	
	Hazard ratio (95% confidence interval)	p-value	Hazard ratio (95% confidence interval)	p-value
Low stable (reference)	**–**		**–**	
Medium increasing	2.68 (1.29, 5.58)	0.008	3.83 (1.76, 8.311)	0.001
High increasing	5.67 (2.76, 11.68)	<0.001	6.97 (3.00, 16.16)	<0.001
Very high increasing	13.27 (6.42, 27.42)	<0.001	14.22 (5.92, 34.17)	<0.001
Menopausal status	–	–		
Pre-menopausal (reference)			–	–
Early perimenopause			1.23 (0.80, 1.88)	0.348
Race/ethnicity	–	–		
Black/African American			1.34 (0.81, 2.21)	0.253
Chinese/Chinese American			3.48 (1.60, 7.59)	0.002
Japanese/Japanese American			3.04 (1.32, 7.01)	0.009
Caucasian/White Non-Hispanic (reference)			–	–
Education	–	–		
Less than high school			3.44 (1.23, 9.60)	0.019
High school			2.66 (1.30, 5.43)	0.007
Some college/technical			1.48 (0.75, 2.91)	0.253
College graduate			0.85 (0.38, 1.91)	0.691
Postgraduate (reference)			–	–
Total family income	–	–		
Less than $19,999			1.78 (0.68, 4.67)	0.244
$20,000–49,999			1.14 (0.50, 2.60)	0.752
$50,000–99,999			0.96 (0.43, 2.15)	0.918
$100,000 or more (reference)			–	–
Smoking status	–	–		
Non-smoker (reference)			–	–
Smoker			1.08 (0.61, 1.93)	0.792
Played sports/exercised during past year	–	–		
Never			1.99 (1.02, 3.87)	0.042
Less than once a month			2.79 (1.41, 5.52)	0.003
Once a month			3.21 (1.28, 8.06)	0.013
2–3 times a month			1.47 (0.61, 3.51)	0.388
Once a week			2.12 (0.96, 4.69)	0.065
More than once a week (reference)			–	–
Family History of diabetes	–	–		
No (reference)			–	–
Yes			1.55 (1.00, 2.39)	0.051
Unknown			0.88 (0.27, 2.90)	0.0835

### Trajectory groups of waist circumference

Waist circumference trajectory during the first five visits was best fitted by a four-group model (Fig 3). The estimates of the trajectory parameters and model adequacy information are presented in S2 Table. The first trajectory group (*increasing*) comprised of 35.9% women with a waist circumference that puts them at an increased risk for metabolic complications, and the waist circumference increased gradually but remained within the range of increased risk. The second trajectory group (*normal increasing*) comprised of 38.4% women with a waist circumference that increased slightly but remained within the low risk of metabolic complications category during the waist circumference measurement period. The third trajectory group (*high increasing*) comprised of 18.7% women had a waist circumference of around 100 cm that was at substantially increased risk of metabolic complication at first visit, and slightly increased during the waist circumference measurement period. The fourth trajectory group (*very high stable*) comprised of 6.9% women with a waist circumference of around 120 cm that was at substantially increased risk of metabolic complication at first visit, and remained stable during the subsequent four visits.

**Fig 3:**
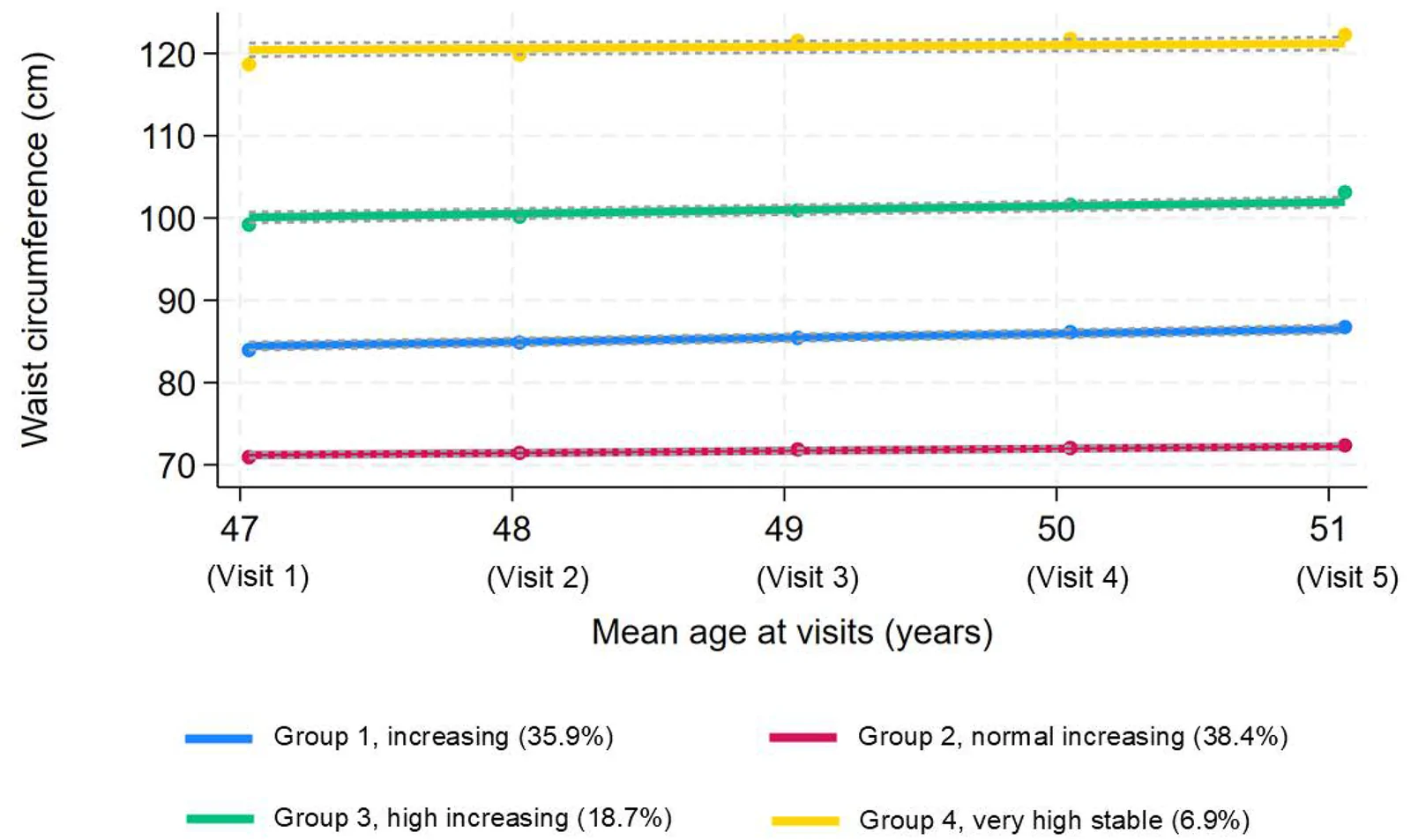
Waist circumference trajectory groups during the measurement period. Solid lines represent the estimated trajectories, dot symbols represent the observed group means at each age, and the dashed lines represent 95% pointwise confidence intervals on the estimated trajectories.

### Baseline characteristics according to waist circumference trajectory groups

The baseline characteristics of the women according to the waist circumference trajectory groups are presented in Table 5. The age of women in the trajectory groups were similar. There were statistically significant differences in menopausal status, race/ethnicity, educational level, total family income, played sports/exercise in past year and family history of diabetes among the trajectory groups. More than half of the women in trajectory groups 1 and 3 were in premenopausal, while more than half of the women in trajectory groups 2, and 4 were in early perimenopause at baseline. Most women in all trajectory groups were Caucasian/White Non-Hispanic or Black/African American. Trajectory group 2 had a lower proportion of Black/African American but a higher proportion of Chinese/Chinese American and Japanese/Japanese American than other trajectory groups. The highest proportion of women in trajectory group 2 had post graduate education, whereas the highest proportion of women in other trajectory groups had some college/technical school level education. The highest proportion of women in trajectory groups 1, 2, and 3 had a total family income of $$50,000 to $99,999, whereas the highest proportion of women in trajectory group 4 had a total family income of 20,000 to $49,999. A higher proportion of women in trajectory group 4 reported that they never played sports or exercised in the past year than women in other trajectory groups. A higher proportion of women in trajectory group 4 had a family history of diabetes mellitus than women in other trajectory groups.

**Table 5:** Baseline characteristics according to waist circumference trajectory groups.

Characteristics	Frequency (%)				P value
	Trajectory group 1, increasing (n = 512)	Trajectory group 2, normal increasing (n = 536)	Trajectory group 3, high increasing (n = 261)	Trajectory group 4, very high stable (n = 97)	
Menopausal status					0.003
Pre-menopausal	292 (57.03)	214 (39.93)	139 (53.26)	48 (49.48)	
Early perimenopause	220 (42.97)	322 (60.07)	122 (46.74)	49 (50.52)	
Age in years					
Mean (SD)	45.84 (2.61)	46.00 (2.63)	45.93 (2.79)	45.80 (2.68)	0.657
Median (IQR)	46 (44, 48)	46 (44, 48)	46 (44, 48)	46 (44, 48)	
Race/ethnicity					<0.001
Black/African American	133 (25.98)	46 (8.58)	103 (39.46)	48 (49.48)	
Chinese/Chinese American	51 (9.96)	87 (16.23)	7 (2.68)	1 (1.03)	
Japanese/Japanese American	43 (8.40)	132 (24.63)	2 (0.77)	0 (0.00)	
Caucasian/White Non-Hispanic	285 (55.66)	271 (50.56)	149 (57.09)	48 (49.48)	
Educational level					<0.001
Less than High	9 (1.76)	16 (2.99)	5 (1.92)	5 (5.15)	
High school graduate	62 (12.11)	58 (10.82)	46 (17.62)	22 (22.68)	
Some college/technical school	165 (32.23)	141 (26.31)	94 (36.02)	36 (37.11)	
College graduate	124 (24.22)	152 (28.36)	47 (18.01)	18 (18.56)	
Post graduate education	152 (29.69)	169 (31.53)	69 (26.44)	16 (16.49)	
Total family income					<0.001
Less than $19,999	28 (5.47)	20 (3.73)	23 (8.81)	22 (22.68)	
$20,000–49,999	164 (32.03)	143 (26.68)	102 (39.08)	42 (43.30)	
$50,000–99,999	214 (41.80)	236 (44.03)	108 (41.38)	29 (29.90)	
$100,000 or more	106 (20.70)	137 (25.56)	28 (10.73)	4 (4.12)	
Smoking status					0.132
Not a current smoker	452 (88.28)	490 (91.42)	226 (86.59)	84 (86.60)	
Current smoker	60 (11.72)	46 (8.58)	35 (13.41)	13 (13.40)	
Played sports/exercised during past year					<0.001
Never	90 (17.58)	71 (13.25)	48 (18.39)	45 (46.39)	
Less than once a month	61 (11.91)	55 (10.26)	52 (19.92)	14 (14.43)	
Once a month	32 (6.25)	23 (4.29)	10 (3.83)	6 (6.19)	
2–3 times a month	69 (13.48)	43 (8.02)	43 (16.48)	12 (12.37)	
Once a week	57 (11.13)	62 (11.57)	38 (14.56)	7 (7.22)	
More than once a week	203 (39.65)	282 (52.61)	70 (26.82)	13 (13.40)	
Family History of diabetes mellitus					0.008
No	327 (63.87)	354 (66.04)	159 (60.92)	44 (45.36)	
Yes	166 (32.42)	161 (30.04)	95 (36.40)	47 (48.45)	
Unknown	19 (3.71)	21 (3.92)	7 (2.68)	6 (6.19)	

SD, standard deviation; IQR, interquartile range.

### Association between waist circumference trajectory groups and incident diabetes mellitus

Among 90 incident diabetes, 20 developed in the increasing trajectory group (4.51 cases per 1000 person-years), 12 (13.3%) in the normal increasing trajectory group, 35 (38.9%) in the high increasing trajectory group, and 23 (25.6%) in the very high stable trajectory group. The adjusted HRs for diabetes compared to the normal increasing group were 2.26 (95% CI: 1.06, 4.85) in increasing, 8.12 (95% CI: 3.64, 18.11) in high increasing, and 11.30 (95% CI: 4.69, 27.25) in very high stable trajectory groups (Table 6).

**Table 6:** Association between waist circumference trajectory groups and incident diabetes.

Trajectory group	Unadjusted		Adjusted	
	Hazard ratio (95% confidence interval)	p-value	Hazard ratio (95% confidence interval)	p-value
Normal increasing (reference)	**–**		**–**	
Increasing	1.76 (0.86, 3.59)	0.123	2.26 (1.06, 4.85)	0.036
High increasing	6.35 (3.30, 12.23)	<0.001	8.12 (3.64, 18.11)	<0.001
Very high stable	11.73 (5.84, 23.59)	<0.001	11.30 (4.69, 27.25)	<0.001
Menopausal status	–	–		
Pre-menopausal (reference)			–	–
Early perimenopause			1.06 (0.69, 1.62)	0.793
Race/ethnicity	–	–		
Black/African American			1.37 (0.83, 2.26)	0.224
Chinese/Chinese American			2.98 (1.38, 6.42)	0.005
Japanese/Japanese American			3.60 (1.48, 8.73)	0.005
Caucasian/White Non-Hispanic (reference)			–	–
Education	–	–		
Less than high school			3.21 (1.13, 9.13)	0.028
High school			2.52 (1.22, 5.17)	0.012
Some college/technical			1.48 (0.75, 2.91)	0.255
College graduate			0.83 (0.37, 1.87)	0.645
Postgraduate (reference)			–	–
Total family income	–	–		
Less than $19,999			1.65 (0.62, 4.40)	0.318
$20,000–49,999			1.10 (0.48, 2.52)	0.814
$50,000–99,999			0.92 (0.41, 2.06)	0.833
$100,000 or more (reference)			–	–
Smoking status	–	–		
Non-smoker (reference)			–	–
Smoker			0.98 (0.55, 1.75)	0.956
Played sports/exercised during past year	–	–		
Never			1.92 (0.97, 3.78)	0.060
Less than once a month			2.72 (1.38, 5.36)	0.004
Once a month			2.98 (1.18, 7.51)	0.021
2–3 times a month			1.35 (0.57, 3.24)	0.495
Once a week			2.06 (0.93, 4.55)	0.075
More than once a week (reference)			–	–
Family History of diabetes	–	–		
No (reference)			–	–
Yes			1.57 (1.02, 2.43)	0.042
Unknown			0.97 (0.30, 3.17)	0.957

### Multi-trajectories of BMI and waist circumference and their association with incidence diabetes

Four multi-trajectories of BMI and waist circumference were identified where the women followed similar trajectories of BMI and waist circumference (Fig 4). Group 1 comprised of 490 (34.5%) women with high increasing BMI and increasing waist circumference trajectories. Group 2 comprised of 461 (33.1%) women with low stable BMI and normal increasing waist circumference trajectories. Group 3 comprised of 310 (22.1%) women with medium increasing BMI and high increasing waist circumference trajectories. Group 4 comprised of 145 (10.3%) women with very high increasing BMI and very high stable waist circumference trajectories. Among 90 incident diabetes, 21 developed in group 1 (8.69 cases per 1000 person-years), 9 developed in group 2 (3.93 cases per 1000 person-years), 29 developed in group 3 (19.58 cases per 1000 person-years), and 31 developed in group 4 (47.47 cases per 1000 person-years). The adjusted HRs for diabetes compared to group 2 were 2.95 (95% CI: 1.31, 6.66) in group 1, 5.95 (95% CI: 2.57, 13.76) in group 3, and 11.80 (95% CI: 4.85, 28.72) in group 4 (Table 6) (data not shown).

**Fig 4:**
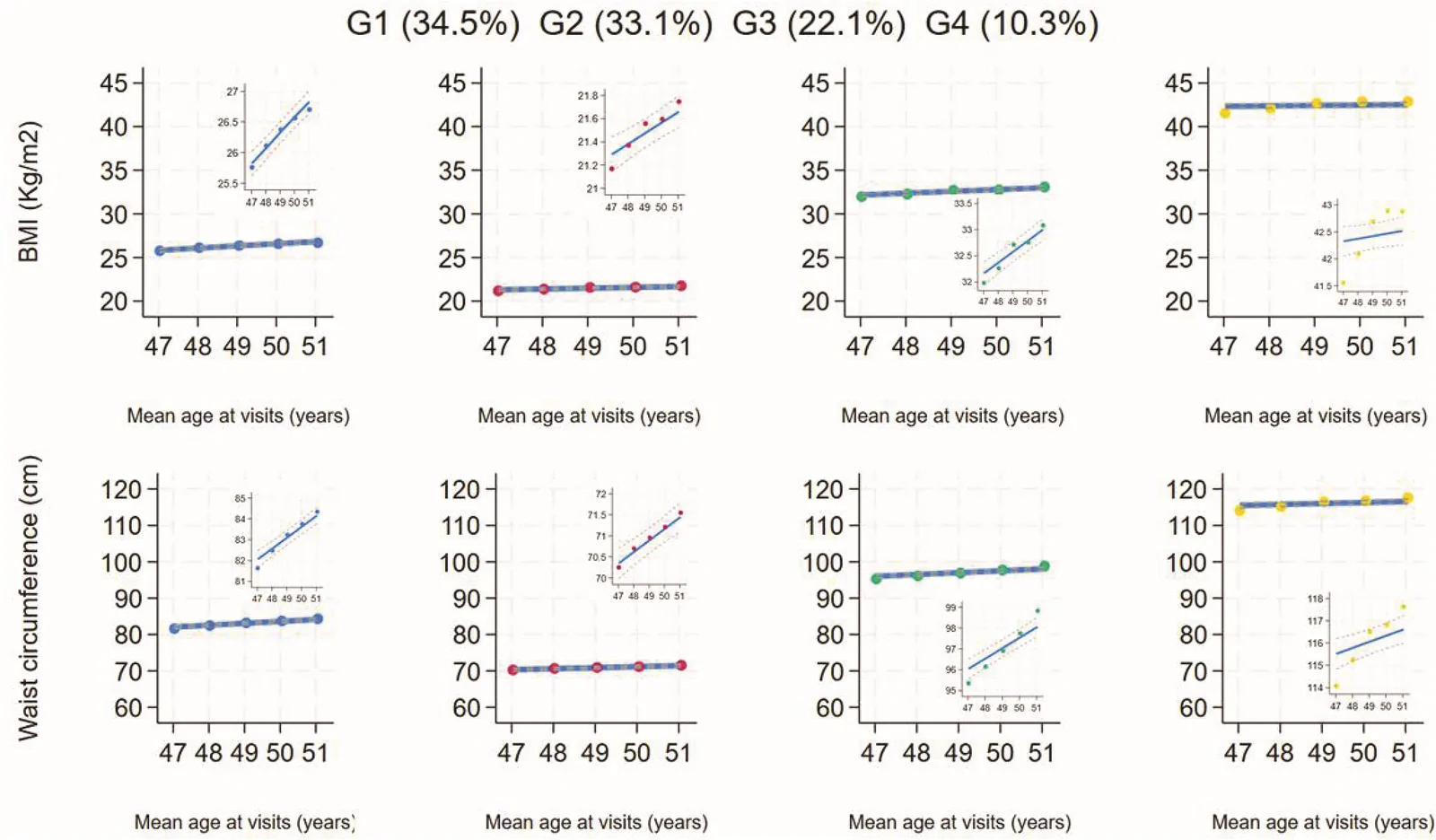
Multi-trajectories of BMI and waist circumference trajectory groups during the measurement period. Solid lines represent the estimated trajectories, dot symbols represent the observed group means at each age, and the dashed lines represent 95% pointwise confidence intervals on the estimated trajectories.

## Discussion

Using longitudinal data from the SWAN, this study sought to identify the BMI and waist circumference trajectory groups, individually and combined (multi-trajectory) and examine their association with the risk of developing diabetes among midlife women in USA. We identified four BMI trajectory groups: medium increasing, low stable, high increasing, and very high increasing. Women belonging to the medium increasing, high increasing and very high increasing BMI trajectory groups had an increased rate of diabetes compared to women belonging to the low stable BMI trajectory group. Four waist circumference trajectory groups were identified: increasing, normal increasing, high increasing, and very high stable. Compared to women in the normal increasing waist circumference trajectory group, women in the other three groups had an increased rate of diabetes. We identified four groups of women who followed similar trajectories of BMI and waist circumference using the multi-trajectory modeling: group 1 (high increasing BMI and increasing waist circumference trajectories), group 2 (low stable BMI and normal increasing waist circumference), group 3 (medium increasing BMI and high increasing waist circumference), and Group 4 (very high increasing BMI and very high stable waist circumference) with an increased rate of diabetes in groups 1, 3, and 4 compared to group 2.

In our study, the four identified BMI trajectory groups all had an increasing trend but with different baseline BMI categories. More than one-third women belonged to the low stable trajectory group who maintained a healthy BMI during the BMI measurement period. Another one-third women had BMI trajectory in the overweight category (medium increasing), and about one-fifth women had class 1 obesity BMI trajectory (high increasing). Approximately eight percent women had class 3 obesity that increased slightly during the BMI measurement period. We found an incremental hazard (3.83, 6.97 and 14.22 times) of diabetes in women in the medium increasing, high increasing and very high increasing BMI trajectory groups, respectively, compared to women in the low stable BMI trajectory group. Our finding of an increasing risk of diabetes in women in the trajectory groups in the overweight and obese BMI categories is consistent with findings from previous studies.

Few previous studies have identified different BMI trajectory groups using group-based trajectory modeling. A study among 4,593 Australian women aged 50–65 years identified four BMI trajectory groups over a 12-year period: stable (72.9%), high stable (19.3%), plateau – decrease (4.5%) and plateau – increase (3.4%) with the stable group having normal BMI and the others with overweight or obese BMI at baseline [34]; a higher proportion of women in this study had stable and normal BMI compared to our study. The incidence of diabetes in that study was lowest in the stable BMI group; incidence percentage (95% CI): 1.9 (1.4–2.6) in stable, 5.7 (4.9–8.2) in increase-plateau, 11.1 (6.8–18.2) in plateau-decrease, and 10.7 (6.6–17.5) in plateau-increase group.

Another study among 12,302 middle aged Australian women followed for an average of 16 years identified seven distinct BMI trajectories among women within different categories of BMI at baseline: Normal weight- low stable (8%), normal weight mid modest increased (21%), normal weight high modest increase (17%), overweight low-rapid increase (30%), overweight high rapid increase (13%), obese low rapid increase (9%), and obese high rapid increase (2%) [9]. Compared to our study, this study had a higher proportion (46%) of women with normal BMI at baseline but most of them had an increasing BMI trajectory, and had a rapid increase in BMI among overweight and obese BMI groups. That study also found significant increased risk of type 2 diabetes mellitus among different trajectory groups compared to the normal weight -low stable group except the normal weight high modest increase group. The increased risk varied among the groups with a range from hazard ratio (HR) = 2.06 (95% CI: 1.38–3.07) in normal weight mid modest increase group to HR = 8.35 (95% CI: 5.59–12.47) in the obese-low rapid increase trajectory group. A study among 6,380 females aged 50−60 years using data from the Health and Retirement Study in the USA identified five BMI trajectory groups: stable (6%), rapidly decreasing (38%), slowly decreasing (7%), slowly increasing (42%) and rapidly increasing (7%); an increased risk of diabetes was observed in the increasing trajectory group compared to the stable trajectory group (adjusted risk ratio, ARR = 1.18, 95% CI: 1.00–1.38) [35].

For the waist circumference trajectories, a study among 2,650 middle aged Korean women using data from the Korean genome and epidemiology study identified five trajectory groups: very low-stable, low-stable, moderate-stable, elevated-increasing, and high-increasing. All trajectory groups had significantly higher risk of diabetes compared to very low-stable group. However, the risks of diabetes were no longer higher after adjusting for BMI [14]. Nevertheless, in our study, we identified four groups of women who followed similar trajectories of BMI and waist circumference, and found that the rate of diabetes was higher in the groups where the BMI and waist circumference trajectories were above the healthy weight vs. healthy weight category or above the normal risk vs. normal risk level, respectively. Further studies are warranted to better understand the interrelation between the two trajectories.

A growing body of evidence suggests that sustained weight loss from overweight or obesity to healthy weight during midlife is associated with long-term health benefits such as a decreased risk of chronic diseases including diabetes and all-cause mortality. Midlife women aged 40–60 years with normal or overweight BMI are recommended to maintain weight or limit weight gain to prevent overweight and gain long-term health benefits [36]. One study found a HR of 0.52 (95% CI: 0.35–0.78) in a cohort followed for a median of 22.8 year, and a HR of 0.43 (95% CI: 0.29–0.66) in another cohort followed for a median of 12.2 years) for developing chronic diseases, including diabetes; similar benefits were observed in women aged <50 years and ≥50 years for incident chronic disease [37]. A HR of 0.81 (95% CI: 0.68–0.96) was found in a cohort followed-up a median of 35 years for all-cause mortality in that study. Another study evaluating a 12-week obesity educator program found significant reduction of weight, waist circumference, improvements in glycemic control, menopausal symptoms and psychological well-being among women during menopausal transition [38]. Thus, midlife provides a critical window to prevent chronic diseases, including diabetes when women are at increased risk of weight gain.

One of the strengths of this study is the use of data from the prospective study SWAN, a longitudinal study that provides a comprehensive, diverse sample of midlife women from various ethnic backgrounds in the USA. This diversity allowed for a more generalizable understanding of the relationship between the trajectories of measures of obesity and incident diabetes in midlife women. The longitudinal nature of the study allowed us to identify BMI and waist circumference trajectory groups during the initial five visits and examine their association with diabetes developed during the subsequent five visits. Additionally, we examined the interrelationship between these two parameters of obesity by identifying clusters of women who followed similar trajectories of BMI and waist circumference, and their association with incident diabetes, which adds to the knowledge base.

There are some limitations to the study. We excluded 1,401 women with missing information on diabetes during the study. However, among them, 811 would have been excluded even if information on diabetes was available because 769 had less than 3 BMI and/or 3 waist circumference records, 7 were pregnant during the study, and 35 developed diabetes during visits 1–5 measurement period. A minimum of 300–500 sample size is considered to be adequate to identify the number of distinctive trajectories and the shape of each trajectory group in group-based trajectory analysis [31]. As such, our sample size of 1,406 women with at least 3 BMI and 3 waist circumference records and information on the outcome incident diabetes is sufficient to identify distinctive trajectories of BMI and waist circumference and to examine their associations with incident diabetes. Only 90 incident diabetes cases were identified during the sixth to tenth visits. The relatively small number of incident diabetes occurring during the follow-up period may have resulted in HR estimates to be imprecise with wide 95% CIs and increased the potential for model overfitting, particularly for covariate categories with few events. Thus, these findings should be interpreted cautiously considering both the magnitude and 95% CIs rather than statistical significance alone. Incident diabetes was ascertained from self-report alone without clinical or laboratory confirmation. As such there remains the potential for biases from misclassification, under-diagnosis, and differential reporting. Information on the type of diabetes (type 1 or type 2) was not available, but it can be assumed that most of the cases of diabetes in this population were type 2. Many assessments were self-reported by the SWAN participants, which has the potential for information bias (notably recall or social desirability bias) leading to under- or over-reporting of different covariates. Although the SWAN study aimed to have a diverse racial cohort, the participants were Non-Hispanic/White (47%), African American (28%), Hispanic (9%), Chinese (8%), and Japanese women (3%) limiting generalizability of underrepresented racial groups. Our study sample did not include any Hispanic participants. As a result, the findings may not generalizable to this ethnic group. Finally, SWAN recruited participants from specific geographic locations, which limits generalizability to the midlife women population in the geographical locations not included in the study such as the southeast.

## Conclusions

Our findings provide further evidence that BMI and waist circumference trajectories individually and clusters of women following similar trajectories of these two obesity parameters above the normal range are significantly associated with an increased rate of diabetes in middle aged pre- and peri-menopausal women. Given the rising prevalence of obesity and type 2 diabetes, these findings underscore the importance of targeted public health strategies and interventions to reduce the burden of diabetes during the critical time in midlife women. Weight management interventions targeting BMI and waist circumference reduction for overweight or obese women, weight maintenance in women within normal BMI range, and maintaining waist circumference within the normal risk level could play a crucial role in reducing the incidence of diabetes in this population. New public health strategies can be developed aimed at encouraging physical activity, a balanced diet, and behavioral modifications during midlife. Future research should assess the effectiveness of different strategies targeting modifiable risk factors in preventing diabetes.

## Supporting information

S1 Table Competing models for selecting trajectory groups of body mass index (BMI) and waist circumference. (DOCX)

S2 Table Trajectory groups of body mass index (BMI) and waist circumference. (DOCX)
