# Supplementary material for: Body mass index and waist circumference trajectories among midlife women in the United States and their association with incident diabetes: The Study of Women’s Health Across the Nation
Source: PLoS One. 2026 Nov 12;21(11):e0357147. doi: 10.1371/journal.pone.0357147 (PMC13759042; doi:10.1371/journal.pone.0357147)
Supplement: Supplementary file 1 [file pone.0357147.s001.docx]

**S1 Table. Competing models for selecting trajectory groups of body mass index (BMI) and waist circumference**

| **Number of trajectory groups** | **BIC** | **Smallest group (%)** |
| --- | --- | --- |
| **BMI trajectory** |  |  |
| 1 | -23135.28 | 100.0 |
| 2 | -20375.81 | 25.4 |
| 3 | -18680.42 | 11.8 |
| 4 | -17794.09 | 8.3 |
| 5 | -16891.82 | 2.1 |
| **Waist circumference** |  |  |
| 1 | -28723.96 | 100.0 |
| 2 | -26091.34 | 29.2 |
| 3 | -24556.59 | 13.7 |
| 4 | -23715.21 | 7.0 |
| 5 | -22995.22 | 2.9 |
