# Supplementary material for: Body mass index and waist circumference trajectories among midlife women in the United States and their association with incident diabetes: The Study of Women’s Health Across the Nation
Source: PLoS One. 2026 Nov 12;21(11):e0357147. doi: 10.1371/journal.pone.0357147 (PMC13759042; doi:10.1371/journal.pone.0357147)
Supplement: Supplementary file 2 [file pone.0357147.s002.docx]

**S2 Table. Trajectory groups of body mass index (BMI) and waist circumference**

| **Group** | **Model estimated group membership, %** | **Assigned group**  **membership using**  **maximum**  **probability rule, n (%)** | **Average posterior**  **probability** | **Odds of correct**  **classification** | **Parameter** | **Estimate (SE)** | ***t* statistic** | **BIC** |
| --- | --- | --- | --- | --- | --- | --- | --- | --- |
| **BMI trajectory** | | | | | | | | |
| High increasing | 19.5 | 271 (19.3) | 0.98 | 193.43 | Intercept | -419.93 (208.66) | -2.01 | -17779.28 |
|  |  |  |  |  | Linear | 27.42 (12.74) | 2.15 |  |
|  |  |  |  |  | Quadratic | -0.56 (0.26) | -2.15 |  |
|  |  |  |  |  | Cubic | 0.00 (0.00) | 2.18 |  |
| Low stable | 37.4 | 524 (37.4) | 0.97 | 50.52 | Intercept | 16.61 (0.98) | 17.00 |  |
|  |  |  |  |  | Linear | 0.10 (0.02) | 5.27 |  |
| Medium increasing | 34.8 | 494 (35.1) | 0.95 | 33.60 | Intercept | 12.06 (1.13) | 10.64 |  |
|  |  |  |  |  | Linear | 0.31 (0.02) | 13.12 |  |
| Very high increasing | 8.3 | 117 (8.3) | 0.98 | 576.57 | Intercept | 38.81 (2.06) | 18.85 |  |
|  |  |  |  |  | Linear | 0.01 (0.04) | 2.31 |  |
| **Waist circumference trajectory** | | | | | | | | |
| Increasing | 35.9 | 512 (36.4) | 0.95 | 32.94 | Intercept | 60.42 (2.61) | 23.16 | -23694.39 |
|  |  |  |  |  | Linear | 0.51 (0.05) | 9.63 |  |
| Normal increasing | 38.4 | 536 (38.1) | 0.98 | 66.68 | Intercept | 58.79 (2.17) | 27.11 |  |
|  |  |  |  |  | Linear | 0.26 (0.04) | 6.00 |  |
| High increasing | 18.7 | 261 (18.6) | 0.97 | 140.76 | Intercept | 78.23 (3.28) | 23.84 |  |
|  |  |  |  |  | Linear | 0.46 (0.07) | 7.04 |  |
| Very high stable | 6.9 | 97 (6.9) | 0.99 | 1105.05 | Intercept | 111.41 (4.98) | 22.39 |  |
|  |  |  |  |  | Linear | 0.19 (0.10) | 1.91 |  |
