# Supplementary material for: Body mass index and waist circumference trajectories among midlife women in the United States and their association with incident diabetes: The Study of Women’s Health Across the Nation
Source: PLoS One. 2026 Nov 12;21(11):e0357147. doi: 10.1371/journal.pone.0357147 (PMC13759042; doi:10.1371/journal.pone.0357147)
Supplement: Supplementary file 3 [file pone.0357147.s003.docx]

**Reviewer Comments – PONE-D-26-05748**

**Overall Summary**

This manuscript examines body mass index (BMI) and waist circumference (WC) trajectories among midlife women in the SWAN cohort and evaluates their association with incident diabetes. Using group-based trajectory modelling across the first five visits and modified Poisson regression to estimate incidence rate ratios (IRRs) during visits 6–10, the authors identify four trajectory groups for BMI and WC separately, and four joint multi-trajectories. Higher and increasing obesity trajectories were strongly associated with elevated diabetes risk.

The study addresses an important public health question using a well-established longitudinal cohort. The use of trajectory modelling to capture dynamic changes in adiposity is appropriate and potentially informative. However, several methodological and reporting issues require clarification and strengthening before the manuscript can be considered for publication.

**Major Comments**

**1. Ethics Statement – Inconsistent and Potentially Incorrect**

The manuscript states:

“This study used publicly available de-identified secondary data… ethical approval was not needed.”

However:

- SWAN is a human subjects study.
- Even for secondary data analyses, authors must state whether their institution deemed the study exempt or not human subjects research.

The current ethics statement is insufficient and potentially non-compliant with journal policy.

**Recommendation:**
Clarify:

- Whether institutional IRB exemption was obtained.
- Permission to use SWAN study data for manuscript publication

**2. Incident Diabetes Definition – Risk of Misclassification**

Incident diabetes was defined via **self-report only**. No fasting glucose, HbA1c, or medication verification was used.

- The strong IRRs (up to 12.47)
- Only 90 cases

There is risk of misclassification bias, under-diagnosis and differential reporting

**Recommendation:**
Authors should discuss in this limitation

- Discuss potential misclassification more explicitly.

**3. Extremely High IRRs – Concern for Overestimation**

The reported adjusted IRRs are unusually large:

- BMI very high increasing: IRR 12.47 (5.80–26.78)
- WC very high increasing: IRR 9.79
- Multi-trajectory group 4: IRR 10.43

Given:

- Only 90 total diabetes cases
- Small size of very high groups (~8–10%)

There may be sparse data bias.

**Recommendation:**

- Provide number of events per group clearly in a table.
- Consider presenting absolute risks.
- Time-to-event modelling may be more appropriate.

**4. Time-Varying Covariates Not Considered**

All covariates are baseline-only.

Given 10-year follow-up:

- Physical activity
- Smoking
- Income
- Menopausal status

likely changed over time.

This may bias results. Authors should justify baseline-only adjustment.

**Minor Comments**

1. Remove extra spacing and formatting issues throughout the mansucript
2. Abstract:
   - WC group labelled “very high stable” in methods but “very high increasing” in results. There is inconsistent use of terms.
3. Abstract (Conclusion):
   - Authors overstate causality (“could reduce risk”). It’s an observational study, so please rephrase cautiously.
4. Table 1:
   - Hispanic participants = 0. This can be removed as Hispanic might be excluded from your study.
5. Typographical errors:
   - “it’s ability” → “its ability”
   - Double dollar sign in income category
6. References:
   - Website references should include access date.

**Strengths of the Study**

- Use of established longitudinal cohort (SWAN)
- Trajectory-based approach
- Multi-trajectory modeling (advanced method)
- Clear temporal separation between exposure and outcome
- Large sample size for trajectory modeling

**Recommendation to Editor**

This is a potentially publishable manuscript addressing an important topic. However, significant methodological clarifications and additional analyses are required, particularly regarding:

- Ethics compliance
- Selection bias
- Absolute risks
