# Supplementary material for: Body mass index and waist circumference trajectories among midlife women in the United States and their association with incident diabetes: The Study of Women’s Health Across the Nation
Source: PLoS One. 2026 Nov 12;21(11):e0357147. doi: 10.1371/journal.pone.0357147 (PMC13759042; doi:10.1371/journal.pone.0357147)
Supplement: Supplementary file 4 [file pone.0357147.s005.docx]

**Response to reviewers’ comments:**

We thank the Reviewers for their valuable comments and suggestions, and specific recommendations. We have addressed all comments and recommendations and hope that the revisions have improved the clarity and strengthened the paper. Below are point-by-point responses to the comments:

**Reviewer Comments – PONE-D-26-05748**

**Overall Summary**

This manuscript examines body mass index (BMI) and waist circumference (WC) trajectories among midlife women in the SWAN cohort and evaluates their association with incident diabetes. Using group-based trajectory modelling across the first five visits and modified Poisson regression to estimate incidence rate ratios (IRRs) during visits 6–10, the authors identify four trajectory groups for BMI and WC separately, and four joint multi-trajectories. Higher and increasing obesity trajectories were strongly associated with elevated diabetes risk.

The study addresses an important public health question using a well-established longitudinal cohort. The use of trajectory modelling to capture dynamic changes in adiposity is appropriate and potentially informative. However, several methodological and reporting issues require clarification and strengthening before the manuscript can be considered for publication.

**Major Comments**

**1. Ethics Statement – Inconsistent and Potentially Incorrect**

The manuscript states:

“This study used publicly available de-identified secondary data… ethical approval was not needed.”

However:

- SWAN is a human subjects study.
- Even for secondary data analyses, authors must state whether their institution deemed the study exempt or not human subjects research.

The current ethics statement is insufficient and potentially non-compliant with journal policy.

**Recommendation:**
Clarify:

- Whether institutional IRB exemption was obtained.
- Permission to use SWAN study data for manuscript publication

**Response:**

We conducted this study using de-identified publicly available secondary data from the Study of Women’s Health Across the Nation (SWAN) study accessed through the Inter-university Consortium for Political and Social Research (ICPSR). While the original SWAN study is a human subjects study, our study using publicly available de-identified data is not deemed human subjects research and does not require Downstate IRB review and approval. The original study was approved by the Institutional Review Boards at each participating site, and informed consent was obtained from all participants. We have clarified this in our ethics statement in the Methods section of the revised manuscript: “*This study used publicly available de-identified secondary data from the SWAN study accessed through the Inter-university Consortium for Political and Social Research (ICPSR). The original study was approved by the Institutional Review Boards at each participating site, and informed consent was obtained from all participants. Because this study used of publicly available de-identified data, it is deemed not to constitute human subjects research. As such, IRB review and approval was not needed.*” (page 4, lines 80-85 in the revised marked manuscript)

It is permissible to publish manuscripts using the publicly available SWAN study data archived at ICPSR. No formal permission is required for individual studies. It has been stated that: “The SWAN Public Use Datasets provide access to longitudinal data describing the physical, biological, psychological, and social changes that occur during the menopausal transition. Data collected from 3,302 SWAN participants from Baseline through Visit 10 are currently available to the public. Users are able to download datasets in a variety of formats, search variables and view recent publications.” (<https://www.swanstudy.org/swan-research/data-access/#:~:text=SWAN%20researchers%20have%20published%20over,been%20addressed%20by%20SWAN%20Investigators>.)

We have cited individual SWAN study datasets in the methods (references #17-28) in the revised manuscript (page 4, line 79 in the revised marked manuscript).

**2. Incident Diabetes Definition – Risk of Misclassification**

Incident diabetes was defined via **self-report only**. No fasting glucose, HbA1c, or medication verification was used.

- The strong IRRs (up to 12.47)
- Only 90 cases

There is risk of misclassification bias, under-diagnosis and differential reporting

**Recommendation:**
Authors should discuss in this limitation

- Discuss potential misclassification more explicitly.

**Response:**

Thank you for this recommendation. We have explicitly acknowledged this limitation in the limitations of the revised manuscript: “*Incident diabetes was ascertained from self-report alone without clinical or laboratory confirmation. Only 90 cases were identified during sixth to tenth visits. As such there remains the potential for biases from misclassification, under-diagnosis, and differential reporting.*” (page 25, lines 394-397 in the revised marked manuscript)

**3. Extremely High IRRs – Concern for Overestimation**

The reported adjusted IRRs are unusually large:

- BMI very high increasing: IRR 12.47 (5.80–26.78)
- WC very high increasing: IRR 9.79
- Multi-trajectory group 4: IRR 10.43

Given:

- Only 90 total diabetes cases
- Small size of very high groups (~8–10%)

There may be sparse data bias.

**Recommendation:**

- Provide number of events per group clearly in a table.
- Consider presenting absolute risks.
- Time-to-event modelling may be more appropriate.

**Response:**

Thank you for these recommendations. We have added a new table, Table 3 that provides the number of incident diabetes by the trajectory groups and rates per 1000 person-years. We have also conducted time-to-event analysis using Cox proportional hazards regression models in the revised manuscript. We have updated the methods, results and discussion accordingly in the revised manuscript.

**4. Time-Varying Covariates Not Considered**

All covariates are baseline-only.

Given 10-year follow-up:

- Physical activity
- Smoking
- Income
- Menopausal status

likely changed over time.

This may bias results. Authors should justify baseline-only adjustment.

**Response:**

It is customary to use the baseline covariates using trajectory groups from group-based trajectory modelling as the exposure. We have used baseline-only covariates for adjustment for examining the association between the trajectory groups identified during the first 5 years (visits 1-5) and incident diabetes ascertained during the next 5 years (visits 6-10). We acknowledge that variables or factors such as physical activity, smoking, income, and menopausal status may have changed during the outcome ascertaining 5-year period (visits 6-10). However, these variables or factors can not affect the trajectory groups that have been identified using the BMI or weight circumference from the past during visits 1-5. As such, they cannot be confounders in the trajectory groups – incident diabetes association. The baseline covariates can affect both the trajectory groups and incident diabetes and are appropriate to adjust for as confounders.

**Minor Comments**

1. Remove extra spacing and formatting issues throughout the manuscript

**Response:** We have removed extra spacing and tried to adhere to the formatting guidelines provided by the journal. We have inserted figure captions immediately after the first paragraph in which the figure is cited, and inserted tables immediately after the first paragraph in which they are cited.

2. Abstract:
   - WC group labelled “very high stable” in methods but “very high increasing” in results. There is inconsistent use of terms.

**Response:** Thank you for identifying this inconsistency. The waist circumference group should be labelled as “very high stable” in the abstract as labelled in the results. We have corrected this in the abstract.

3. Abstract (Conclusion):
   - Authors overstate causality (“could reduce risk”). It’s an observational study, so please rephrase cautiously.

**Response:** We have rephrased the concluding sentence in the abstract avoiding causality. The revised statement is “Public health interventions on weight management could help reduce the burden of diabetes in this population.” (page 2, lines 26-27 in the revised marked manuscript)

4. Table 1:
   - Hispanic participants = 0. This can be removed as Hispanic might be excluded from your study.

**Response:** We have removed the “Hispanic” category from Table 1.

5. Typographical errors:
   - “it’s ability” → “its ability”
   - Double dollar sign in income category

**Response:** We have corrected “it’s ability” to “its ability”, and removed the double dollar sign in income categories in the tables.

6. References:
   - Website references should include access date.

**Response:** We have added the citation date for the website references according to the Vancouver style.

**Strengths of the Study**

- Use of established longitudinal cohort (SWAN)
- Trajectory-based approach
- Multi-trajectory modeling (advanced method)
- Clear temporal separation between exposure and outcome
- Large sample size for trajectory modeling

**Recommendation to Editor**

This is a potentially publishable manuscript addressing an important topic. However, significant methodological clarifications and additional analyses are required, particularly regarding:

- Ethics compliance
- Selection bias
- Absolute risks

**Response:**

We have addressed these issues in our revised manuscript. Please see our responses above.
