# Supplementary material for: Body mass index and waist circumference trajectories among midlife women in the United States and their association with incident diabetes: The Study of Women’s Health Across the Nation
Source: PLoS One. 2026 Nov 12;21(11):e0357147. doi: 10.1371/journal.pone.0357147 (PMC13759042; doi:10.1371/journal.pone.0357147)
Supplement: Supplementary file 6 [file pone.0357147.s007.docx]

**Response to reviewers’ comments:**

We thank the Reviewers for their valuable comments and suggestions. We have addressed all comments and hope that the revisions have further improved the clarity and strengthened the paper. Below are point-by-point responses to the comments:

**Reviewer Comments – PONE-D-26-05748R2**

**Reviewer #2:**

**Comment:** Thank you for your consideration for my comments. I confirmed that the authors respond my suggestions. I have no additional comments.

**Response:** We thank Reviewer #2.

**Reviewer #3:**

**Comment 1:** The authors primarily rely on BIC, a minimum group size of 5%, and posterior-probability diagnostics. It would be useful to report the BIC values for the competing models and briefly explain how clinical interpretability and trajectory separation contributed to the final four-group solution. This would make the model-selection process more transparent and reproducible.

**Response:** Thank you for this helpful comment. We have clarified the model-selection process in the Methods and added the BIC values for the competing models in Supplementary S1 Table. We have added the following in the Methods: “Models The model with the different numbers of trajectory groups were compared using the lowest Bayesian Information Criterion (BIC) as a measure of goodness-of-fit, where the groups with the requirement that each group included at least 5% of the study participants was selected. BIC values for the competing models are presented in S1 Table. The four-group model was selected based on the combined consideration of BIC, minimum group size, trajectory separation, and clinical interpretability. Compared with the three-group model, the four-group model distinguished two clearly separated intermediate BMI trajectories that were combined into a single trajectory in the three-group model. The four trajectories also yielded four clinically distinguishable BMI trajectories corresponding approximately to healthy weight, overweight, class 1 obesity and class 3 obesity with all groups exceeding the 5% minimum group size. The five-group model included an additional trajectory group consisting of only 2.1% of participants below the prespecified 5% minimum group size.” (lines 125-136 in the revised marked manuscript)

**Comment 2:** The relatively small number of diabetes events deserves more attention when interpreting the adjusted estimates. Only 90 incident diabetes cases occurred, while several categorical covariates and four-level trajectory variables are included in the adjusted models. Some estimates, particularly for the smaller trajectory groups, have relatively wide confidence intervals. The authors should acknowledge the possibility of limited precision and potential overfitting, and emphasize the confidence intervals rather than relying primarily on statistical significance.

**Response:** We thank the reviewer for highlighting the relatively small number of incident diabetes events and its implications for the adjusted estimates. We agree that this needs to be acknowledged in the limitations of the study. We have added the following in the limitations of the Discussion: “Only 90 incident diabetes cases were identified during the sixth to tenth visits. The relatively small number of incident diabetes occurring during the follow-up period may have resulted in HR estimates to be imprecise with wide 95% CIs and increased the potential for model overfitting, particularly for covariate categories with few events. Thus, these findings should be interpreted cautiously considering both the magnitude and 95% CIs rather than statistical significance alone.” (lines 377-382 in the revised marked manuscript)

**Comment 3:** The adjustment strategy could be better justified from a clinical and causal perspective. In particular, menopausal status is included as a baseline covariate, although BMI and WC trajectories are themselves closely related to the menopausal transition. Please provide a brief rationale for the covariates included in the final models and clarify whether the intention was to estimate an association independent of baseline menopausal status or to account for potential confounding.

**Response:** We thank the reviewer for this comment. We agree that the rationale for the covariate adjustment strategy should be clarified. The covariates included in the final models were selected a priori based on their clinical relevance to both adiposity trajectories and diabetes risk and their potential to account for baseline differences between trajectory groups. Menopausal status was included as a baseline covariate because menopausal status is clinically related to changes in body composition and adiposity as well as to diabetes risk. Our intention was to account for potential differences in diabetes risk associated with baseline menopausal status and thereby estimate the association between BMI and waist circumference trajectory group and incident diabetes conditional on baseline menopausal status. We recognize that the menopausal transition may itself contribute to changes in BMI and waist circumference over time. We have added the following to the Methods: “These covariates were selected a priori based on their clinical relevance to adiposity and diabetes risk, and their potential to account for baseline differences between adiposity trajectory groups. Baseline menopausal status was included to account for differences in adiposity patterns and diabetes risk associated with menopausal status.” (lines 151-155 in the revised marked manuscript)

**Comment 4:** Some of the clinical terminology regarding menopause should be revised for accuracy. The statement that natural menopause “occurs in every woman between 45 and 55 years” is somewhat too categorical. Menopause is clinically defined retrospectively after 12 consecutive months of amenorrhea without another cause, and the age range is not restricted to 45–55 years. Since menopausal status is also an adjustment variable in the analysis, a more precise definition would improve the clinical accuracy of the manuscript.

**Response:** Thank you for this helpful comment. We agree that the original statement in the Introduction that natural menopause “occurs in every woman between 45 and 55 years” was overly categorical and did not accurately reflect the clinical definition of menopause. We have revised the statement and added the following: “Menopause is the biological transition that naturally occurs in every woman’s life at the end of reproductive years. Natural menopause is clinically defined retrospectively after 12 consecutive months of and is characterized by the cessation of menstruation due to decreased ovarian function in absence of any other physiological or pathological cause, or clinical intervention [4].” (lines 33-36 in the revised marked manuscript). We have also added the definition of premenopausal and early perimenopausal status in the Methods in lines 83-85 in the revised marked manuscript.
