# Supplementary material for: Body mass index and waist circumference trajectories among midlife women in the United States and their association with incident diabetes: The Study of Women’s Health Across the Nation
Source: PLoS One. 2026 Nov 12;21(11):e0357147. doi: 10.1371/journal.pone.0357147 (PMC13759042; doi:10.1371/journal.pone.0357147)
Supplement: Peer review history

# Peer review: Decision Letter 0

**DOI:** 10.1371/journal.pone.0357147.r001

**Authors:** Guoying Wang

5 Mar 2026

Dear Dr. Brewster,

Thank you for submitting your manuscript to PLOS ONE. After careful consideration, we feel that it has merit but does not fully meet PLOS ONE’s publication criteria as it currently stands. Therefore, we invite you to submit a revised version of the manuscript that addresses the points raised during the review process.

Please carefully address the reviewers’ comments. In particular, please address the potential misclassification of diabetes. In addition, the wide 95% confidence intervals suggest that the number of cases in each group may be small. Please provide the number of cases for each group in Table 5.

Please submit your revised manuscript by Apr 19 2026 11:59PM. If you will need more time than this to complete your revisions, please reply to this message or contact the journal office at plosone@plos.org. When you're ready to submit your revision, log on to https://www.editorialmanager.com/pone/ and select the 'Submissions Needing Revision' folder to locate your manuscript file.

- A letter that responds to each point raised by the academic editor and reviewer(s). You should upload this letter as a separate file labeled 'Response to Reviewers'.
- A marked-up copy of your manuscript that highlights changes made to the original version. You should upload this as a separate file labeled 'Revised Manuscript with Track Changes'.
- An unmarked version of your revised paper without tracked changes. You should upload this as a separate file labeled 'Manuscript'.

If you would like to make changes to your financial disclosure, please include your updated statement in your cover letter. Guidelines for resubmitting your figure files are available below the reviewer comments at the end of this letter.

If applicable, we recommend that you deposit your laboratory protocols in protocols.io to enhance the reproducibility of your results. Protocols.io assigns your protocol its own identifier (DOI) so that it can be cited independently in the future. For instructions see: https://journals.plos.org/plosone/s/submission-guidelines#loc-laboratory-protocols. Additionally, PLOS ONE offers an option for publishing peer-reviewed Lab Protocol articles, which describe protocols hosted on protocols.io. Read more information on sharing protocols at https://plos.org/protocols?utm_medium=editorial-email&utm_source=authorletters&utm_campaign=protocols.

We look forward to receiving your revised manuscript.

Kind regards,

Guoying Wang, MD, PhD

Academic Editor

PLOS One

Journal Requirements:

When submitting your revision, we need you to address these additional requirements.

1. Please ensure that your manuscript meets PLOS ONE's style requirements, including those for file naming. The PLOS ONE style templates can be found at

https://journals.plos.org/plosone/s/file?id=wjVg/PLOSOne_formatting_sample_main_body.pdf and

https://journals.plos.org/plosone/s/file?id=ba62/PLOSOne_formatting_sample_title_authors_affiliations.pdf

2. Thank you for stating the following in the Acknowledgments Section of your manuscript:

“The Study of Women's Health Across the Nation (SWAN) has grant support from the National Institutes of Health (NIH), DHHS, through the National Institute on Aging (NIA), the National Institute of Nursing Research (NINR) and the NIH Office of Research on Women's Health (ORWH) (Grants U01NR004061; U01AG012505, U01AG012535, U01AG012531, U01AG012539, U01AG012546, U01AG012553, U01AG012554, U01AG012495, and U19AG063720). The content of this paper is solely the responsibility of the authors and does not necessarily represent the official views of the NIA, NINR, ORWH, or the NIH. This study did not receive any funding.”

We note that you have provided funding information that is not currently declared in your Funding Statement. However, funding information should not appear in the Acknowledgments section or other areas of your manuscript. We will only publish funding information present in the Funding Statement section of the online submission form.

Please remove any funding-related text from the manuscript and let us know how you would like to update your Funding Statement. Currently, your Funding Statement reads as follows:

“The author(s) received no specific funding for this work.”

Please include your amended statements within your cover letter; we will change the online submission form on your behalf.

3. Thank you for stating the following financial disclosure:

“The Study of Women's Health Across the Nation (SWAN) has grant support from the National Institutes of Health (NIH), DHHS, through the National Institute on Aging (NIA), the National Institute of Nursing Research (NINR) and the NIH Office of Research on Women's Health (ORWH) (Grants U01NR004061; U01AG012505, U01AG012535, U01AG012531, U01AG012539, U01AG012546, U01AG012553, U01AG012554, U01AG012495, and U19AG063720). The content of this paper is solely the responsibility of the authors and does not necessarily represent the official views of the NIA, NINR, ORWH, or the NIH. This study did not receive any funding.”

At this time, please address the following queries:

a) Please clarify the sources of funding (financial or material support) for your study. List the grants or organizations that supported your study, including funding received from your institution.

b) State what role the funders took in the study. If the funders had no role in your study, please state: “The funders had no role in study design, data collection and analysis, decision to publish, or preparation of the manuscript.”

c) If any authors received a salary from any of your funders, please state which authors and which funders.

d) If you did not receive any funding for this study, please state: “The authors received no specific funding for this work.”

Please include your amended statements within your cover letter; we will change the online submission form on your behalf.

4. Please provide a complete Data Availability Statement in the submission form, ensuring you include all necessary access information or a reason for why you are unable to make your data freely accessible. If your research concerns only data provided within your submission, please write "All data are in the manuscript and/or supporting information files" as your Data Availability Statement.

5. Please amend the manuscript submission data (via Edit Submission) to include author Sharifa Nasreen.

6. We note that you have included the phrase “data not shown” in your manuscript. Unfortunately, this does not meet our data sharing requirements. PLOS does not permit references to inaccessible data. We require that authors provide all relevant data within the paper, Supporting Information files, or in an acceptable, public repository. Please add a citation to support this phrase or upload the data that corresponds with these findings to a stable repository (such as Figshare or Dryad) and provide and URLs, DOIs, or accession numbers that may be used to access these data. Or, if the data are not a core part of the research being presented in your study, we ask that you remove the phrase that refers to these data.

7. We notice that your supplementary table is uploaded with the file type 'Figure'. Please amend the file type to 'Supporting Information'. Please ensure that each Supporting Information file has a legend listed in the manuscript after the references list.

8. If the reviewer comments include a recommendation to cite specific previously published works, please review and evaluate these publications to determine whether they are relevant and should be cited. There is no requirement to cite these works unless the editor has indicated otherwise.

[Note: HTML markup is below. Please do not edit.]

Reviewers' comments:

Reviewer's Responses to Questions

**Comments to the Author**

1. Is the manuscript technically sound, and do the data support the conclusions?

Reviewer #1: Yes

Reviewer #2: Yes

**********

2. Has the statistical analysis been performed appropriately and rigorously?

Reviewer #1: I Don't Know

Reviewer #2: Yes

**********

3. Have the authors made all data underlying the findings in their manuscript fully available?

The PLOS Data policy

Reviewer #1: Yes

Reviewer #2: Yes

**********

4. Is the manuscript presented in an intelligible fashion and written in standard English?

Reviewer #1: Yes

Reviewer #2: Yes

**********

Reviewer #1: Reviewer Comments – PONE-D-26-05748

Overall Summary

This manuscript examines body mass index (BMI) and waist circumference (WC) trajectories among midlife women in the SWAN cohort and evaluates their association with incident diabetes. Using group-based trajectory modelling across the first five visits and modified Poisson regression to estimate incidence rate ratios (IRRs) during visits 6–10, the authors identify four trajectory groups for BMI and WC separately, and four joint multi-trajectories. Higher and increasing obesity trajectories were strongly associated with elevated diabetes risk.

The study addresses an important public health question using a well-established longitudinal cohort. The use of trajectory modelling to capture dynamic changes in adiposity is appropriate and potentially informative. However, several methodological and reporting issues require clarification and strengthening before the manuscript can be considered for publication.

Major Comments

1. Ethics Statement – Inconsistent and Potentially Incorrect

The manuscript states:

“This study used publicly available de-identified secondary data… ethical approval was not needed.”

However:

• SWAN is a human subjects study.

• Even for secondary data analyses, authors must state whether their institution deemed the study exempt or not human subjects research.

The current ethics statement is insufficient and potentially non-compliant with journal policy.

Recommendation:

Clarify:

• Whether institutional IRB exemption was obtained.

• Permission to use SWAN study data for manuscript publication

2. Incident Diabetes Definition – Risk of Misclassification

Incident diabetes was defined via self-report only. No fasting glucose, HbA1c, or medication verification was used.

• The strong IRRs (up to 12.47)

• Only 90 cases

There is risk of misclassification bias, under-diagnosis and differential reporting

Recommendation:

Authors should discuss in this limitation

• Discuss potential misclassification more explicitly.

3. Extremely High IRRs – Concern for Overestimation

The reported adjusted IRRs are unusually large:

• BMI very high increasing: IRR 12.47 (5.80–26.78)

• WC very high increasing: IRR 9.79

• Multi-trajectory group 4: IRR 10.43

Given:

• Only 90 total diabetes cases

• Small size of very high groups (~8–10%)

There may be sparse data bias.

Recommendation:

• Provide number of events per group clearly in a table.

• Consider presenting absolute risks.

• Time-to-event modelling may be more appropriate.

4. Time-Varying Covariates Not Considered

All covariates are baseline-only.

Given 10-year follow-up:

• Physical activity

• Smoking

• Income

• Menopausal status

likely changed over time.

This may bias results. Authors should justify baseline-only adjustment.

Minor Comments

1. Remove extra spacing and formatting issues throughout the mansucript

2. Abstract:

o WC group labelled “very high stable” in methods but “very high increasing” in results. There is inconsistent use of terms.

3. Abstract (Conclusion):

o Authors overstate causality (“could reduce risk”). It’s an observational study, so please rephrase cautiously.

4. Table 1:

o Hispanic participants = 0. This can be removed as Hispanic might be excluded from your study.

5. Typographical errors:

o “it’s ability” → “its ability”

o Double dollar sign in income category

6. References:

o Website references should include access date.

Strengths of the Study

• Use of established longitudinal cohort (SWAN)

• Trajectory-based approach

• Multi-trajectory modeling (advanced method)

• Clear temporal separation between exposure and outcome

• Large sample size for trajectory modeling

Recommendation to Editor

This is a potentially publishable manuscript addressing an important topic. However, significant methodological clarifications and additional analyses are required, particularly regarding:

• Ethics compliance

• Selection bias

• Absolute risks

Reviewer #2: This study provides a comprehensive longitudinal analysis of BMI and waist circumference trajectories among midlife women in the US, focusing on their distinct associations with incident diabetes. By utilizing a multi-trajectory modeling approach with the SWAN dataset, the authors effectively highlight how the progression of adiposity during the menopausal transition serves as a critical predictor of metabolic risk. The findings underscore the clinical importance of early weight management and targeted public health interventions during this specific life stage to reduce the future burden of diabetes.

While the study provides valuable insights, I have two suggestions to further strengthen the validity of the analysis and its clinical implications.

First, I suggest that the authors provide the correlation between BMI and waist circumference. While it is theoretically possible for an individual to have a high BMI but a low waist circumference, demonstrating a strong correlation would justify the use of the four multi-trajectory groups and confirm that these groups adequately cover the majority of the study population.

Second, I suggest that the authors further expand the Discussion section by citing additional literature to highlight the long-term impact of interventions during midlife. While I understand the rationale for focusing on the menopausal transition, one could argue that interventions aimed at preventing BMI increases prior to midlife might yield even greater effects on reducing incident diabetes. By discussing the specific magnitude of the impact that midlife interventions can have on long-term health outcomes, the authors would significantly strengthen the clinical significance and research contribution of this study.

**********

what does this mean?). If published, this will include your full peer review and any attached files.

If you choose “no”, your identity will remain anonymous but your review may still be made public.

**Do you want your identity to be public for this peer review?**  For information about this choice, including consent withdrawal, please see our Privacy Policy

Reviewer #1: No

Reviewer #2: No

**********

[NOTE: If reviewer comments were submitted as an attachment file, they will be attached to this email and accessible via the submission site. Please log into your account, locate the manuscript record, and check for the action link "View Attachments". If this link does not appear, there are no attachment files.]

To ensure your figures meet our technical requirements, please review our figure guidelines: https://journals.plos.org/plosone/s/figures

You may also use PLOS’s free figure tool, NAAS, to help you prepare publication quality figures: https://journals.plos.org/plosone/s/figures#loc-tools-for-figure-preparation.

NAAS will assess whether your figures meet our technical requirements by comparing each figure against our figure specifications.

Attachment Submitted filename: Reviewer Comments_24.02.2026.docx

# Author comment: Author response to Decision Letter 1

**DOI:** 10.1371/journal.pone.0357147.r002

20 Apr 2026

Response to reviewers’ comments:

We thank the Reviewers for their valuable comments and suggestions, and specific recommendations. We have addressed all comments and recommendations and hope that the revisions have improved the clarity and strengthened the paper. Below are point-by-point responses to the comments:

Reviewer Comments – PONE-D-26-05748

Overall Summary

This manuscript examines body mass index (BMI) and waist circumference (WC) trajectories among midlife women in the SWAN cohort and evaluates their association with incident diabetes. Using group-based trajectory modelling across the first five visits and modified Poisson regression to estimate incidence rate ratios (IRRs) during visits 6–10, the authors identify four trajectory groups for BMI and WC separately, and four joint multi-trajectories. Higher and increasing obesity trajectories were strongly associated with elevated diabetes risk.

The study addresses an important public health question using a well-established longitudinal cohort. The use of trajectory modelling to capture dynamic changes in adiposity is appropriate and potentially informative. However, several methodological and reporting issues require clarification and strengthening before the manuscript can be considered for publication.

Major Comments

1. Ethics Statement – Inconsistent and Potentially Incorrect

The manuscript states:

“This study used publicly available de-identified secondary data… ethical approval was not needed.”

However:

• SWAN is a human subjects study.

• Even for secondary data analyses, authors must state whether their institution deemed the study exempt or not human subjects research.

The current ethics statement is insufficient and potentially non-compliant with journal policy.

Recommendation:

Clarify:

• Whether institutional IRB exemption was obtained.

• Permission to use SWAN study data for manuscript publication

Response:

We conducted this study using de-identified publicly available secondary data from the Study of Women’s Health Across the Nation (SWAN) study accessed through the Inter-university Consortium for Political and Social Research (ICPSR). While the original SWAN study is a human subjects study, our study using publicly available de-identified data is not deemed human subjects research and does not require Downstate IRB review and approval. The original study was approved by the Institutional Review Boards at each participating site, and informed consent was obtained from all participants. We have clarified this in our ethics statement in the Methods section of the revised manuscript: “This study used publicly available de-identified secondary data from the SWAN study accessed through the Inter-university Consortium for Political and Social Research (ICPSR). The original study was approved by the Institutional Review Boards at each participating site, and informed consent was obtained from all participants. Because this study used of publicly available de-identified data, it is deemed not to constitute human subjects research. As such, IRB review and approval was not needed.” (page 4, lines 80-85 in the revised marked manuscript)

It is permissible to publish manuscripts using the publicly available SWAN study data archived at ICPSR. No formal permission is required for individual studies. It has been stated that: “The SWAN Public Use Datasets provide access to longitudinal data describing the physical, biological, psychological, and social changes that occur during the menopausal transition. Data collected from 3,302 SWAN participants from Baseline through Visit 10 are currently available to the public. Users are able to download datasets in a variety of formats, search variables and view recent publications.” (https://www.swanstudy.org/swan-research/data-access/#:~:text=SWAN%20researchers%20have%20published%20over,been%20addressed%20by%20SWAN%20Investigators.)

We have cited individual SWAN study datasets in the methods (references #17-28) in the revised manuscript (page 4, line 79 in the revised marked manuscript).

2. Incident Diabetes Definition – Risk of Misclassification

Incident diabetes was defined via self-report only. No fasting glucose, HbA1c, or medication verification was used.

• The strong IRRs (up to 12.47)

• Only 90 cases

There is risk of misclassification bias, under-diagnosis and differential reporting

Recommendation:

Authors should discuss in this limitation

• Discuss potential misclassification more explicitly.

Response:

Thank you for this recommendation. We have explicitly acknowledged this limitation in the limitations of the revised manuscript: “Incident diabetes was ascertained from self-report alone without clinical or laboratory confirmation. Only 90 cases were identified during sixth to tenth visits. As such there remains the potential for biases from misclassification, under-diagnosis, and differential reporting.” (page 25, lines 394-397 in the revised marked manuscript)

3. Extremely High IRRs – Concern for Overestimation

The reported adjusted IRRs are unusually large:

• BMI very high increasing: IRR 12.47 (5.80–26.78)

• WC very high increasing: IRR 9.79

• Multi-trajectory group 4: IRR 10.43

Given:

• Only 90 total diabetes cases

• Small size of very high groups (~8–10%)

There may be sparse data bias.

Recommendation:

• Provide number of events per group clearly in a table.

• Consider presenting absolute risks.

• Time-to-event modelling may be more appropriate.

Response:

Thank you for these recommendations. We have added a new table, Table 3 that provides the number of incident diabetes by the trajectory groups and rates per 1000 person-years. We have also conducted time-to-event analysis using Cox proportional hazards regression models in the revised manuscript. We have updated the methods, results and discussion accordingly in the revised manuscript.

4. Time-Varying Covariates Not Considered

All covariates are baseline-only.

Given 10-year follow-up:

• Physical activity

• Smoking

• Income

• Menopausal status

likely changed over time.

This may bias results. Authors should justify baseline-only adjustment.

Response:

It is customary to use the baseline covariates using trajectory groups from group-based trajectory modelling as the exposure. We have used baseline-only covariates for adjustment for examining the association between the trajectory groups identified during the first 5 years (visits 1-5) and incident diabetes ascertained during the next 5 years (visits 6-10). We acknowledge that variables or factors such as physical activity, smoking, income, and menopausal status may have changed during the outcome ascertaining 5-year period (visits 6-10). However, these variables or factors can not affect the trajectory groups that have been identified using the BMI or weight circumference from the past during visits 1-5. As such, they cannot be confounders in the trajectory groups – incident diabetes association. The baseline covariates can affect both the trajectory groups and incident diabetes and are appropriate to adjust for as confounders.

Minor Comments

1. Remove extra spacing and formatting issues throughout the manuscript

Response: We have removed extra spacing and tried to adhere to the formatting guidelines provided by the journal. We have inserted figure captions immediately after the first paragraph in which the figure is cited, and inserted tables immediately after the first paragraph in which they are cited.

2. Abstract:

o WC group labelled “very high stable” in methods but “very high increasing” in results. There is inconsistent use of terms.

Response: Thank you for identifying this inconsistency. The waist circumference group should be labelled as “very high stable” in the abstract as labelled in the results. We have corrected this in the abstract.

3. Abstract (Conclusion):

o Authors overstate causality (“could reduce risk”). It’s an observational study, so please rephrase cautiously.

Response: We have rephrased the concluding sentence in the abstract avoiding causality. The revised statement is “Public health interventions on weight management could help reduce the burden of diabetes in this population.” (page 2, lines 26-27 in the revised marked manuscript)

4. Table 1:

o Hispanic participants = 0. This can be removed as Hispanic might be excluded from your study.

Response: We have removed the “Hispanic” category from Table 1.

5. Typographical errors:

o “it’s ability” → “its ability”

o Double dollar sign in income category

Response: We have corrected “it’s ability” to “its ability”, and removed the double dollar sign in income categories in the tables.

6. References:

o Website references should include access date.

Response: We have added the citation date for the website references according to the Vancouver style.

Strengths of the Study

• Use of established longitudinal cohort (SWAN)

• Trajectory-based approach

• Multi-trajectory modeling (advanced method)

• Clear temporal separation between exposure and outcome

• Large sample size for trajectory modeling

Recommendation to Editor

This is a potentially publishable manuscript addressing an important topic. However, significant methodological clarifications and additional analyses are required, particularly regarding:

• Ethics compliance

• Selection bias

• Absolute risks

Response:

We have addressed these issues in our revised manuscript. Please see our responses above.

Attachment Submitted filename: Response to Reviewer Comments_2026 04 14.docx

# Editor report: Decision Letter 1

**DOI:** 10.1371/journal.pone.0357147.r003

**Authors:** Heather Macdonald

4 Jun 2026

Dear Dr. Brewster,

Thank you for submitting your manuscript to PLOS ONE. After careful consideration, we feel that it has merit but does not fully meet PLOS ONE’s publication criteria as it currently stands. Therefore, we invite you to submit a revised version of the manuscript that addresses the points raised during the review process.

Please submit your revised manuscript by Jul 19 2026 11:59PM. If you will need more time than this to complete your revisions, please reply to this message or contact the journal office at plosone@plos.org. When you're ready to submit your revision, log on to https://www.editorialmanager.com/pone/ and select the 'Submissions Needing Revision' folder to locate your manuscript file.

- A letter that responds to each point raised by the academic editor and reviewer(s). You should upload this letter as a separate file labeled 'Response to Reviewers'.
- A marked-up copy of your manuscript that highlights changes made to the original version. You should upload this as a separate file labeled 'Revised Manuscript with Track Changes'.
- An unmarked version of your revised paper without tracked changes. You should upload this as a separate file labeled 'Manuscript'.

If you would like to make changes to your financial disclosure, please include your updated statement in your cover letter. Guidelines for resubmitting your figure files are available below the reviewer comments at the end of this letter.

If applicable, we recommend that you deposit your laboratory protocols in protocols.io to enhance the reproducibility of your results. Protocols.io assigns your protocol its own identifier (DOI) so that it can be cited independently in the future. For instructions see: https://journals.plos.org/plosone/s/submission-guidelines#loc-laboratory-protocols. Additionally, PLOS ONE offers an option for publishing peer-reviewed Lab Protocol articles, which describe protocols hosted on protocols.io. Read more information on sharing protocols at https://plos.org/protocols?utm_medium=editorial-email&utm_source=authorletters&utm_campaign=protocols.

As the corresponding author, your ORCID iD is verified in the submission system and will appear in the published article. PLOS supports the use of ORCID, and we encourage all coauthors to register for an ORCID iD and use it as well. Please encourage your coauthors to verify their ORCID iD within the submission system before final acceptance, as unverified ORCID iDs will not appear in the published article. *Only*  the individual author can complete the verification step; PLOS staff *cannot*  verify ORCID iDs on behalf of authors.

We look forward to receiving your revised manuscript.

Kind regards,

Heather Macdonald, Ph.D

Academic Editor

PLOS One

Journal Requirements:

1. If the reviewer comments include a recommendation to cite specific previously published works, please review and evaluate these publications to determine whether they are relevant and should be cited. There is no requirement to cite these works unless the editor has indicated otherwise.

**Additional Editor Comments:**

Please note that I am a new AE on this submission. I note that the authors did not respond to the comments from the 2nd reviewer. Please resubmit the manuscript and address the 2 comments, which I have copied below.

This study provides a comprehensive longitudinal analysis of BMI and waist circumference trajectories among midlife women in the US, focusing on their distinct associations with incident diabetes. By utilizing a multi-trajectory modeling approach with the SWAN dataset, the authors effectively highlight how the progression of adiposity during the menopausal transition serves as a critical predictor of metabolic risk. The findings underscore the clinical importance of early weight management and targeted public health interventions during this specific life stage to reduce the future burden of diabetes.

While the study provides valuable insights, I have two suggestions to further strengthen the validity of the analysis and its clinical implications.

First, I suggest that the authors provide the correlation between BMI and waist circumference. While it is theoretically possible for an individual to have a high BMI but a low waist circumference, demonstrating a strong correlation would justify the use of the four multi-trajectory groups and confirm that these groups adequately cover the majority of the study population.

Second, I suggest that the authors further expand the Discussion section by citing additional literature to highlight the long-term impact of interventions during midlife. While I understand the rationale for focusing on the menopausal transition, one could argue that interventions aimed at preventing BMI increases prior to midlife might yield even greater effects on reducing incident diabetes. By discussing the specific magnitude of the impact that midlife interventions can have on long-term health outcomes, the authors would significantly strengthen the clinical significance and research contribution of this study.

[Note: HTML markup is below. Please do not edit.]

[NOTE: If reviewer comments were submitted as an attachment file, they will be attached to this email and accessible via the submission site. Please log into your account, locate the manuscript record, and check for the action link "View Attachments". If this link does not appear, there are no attachment files.]

To ensure your figures meet our technical requirements, please review our figure guidelines: https://journals.plos.org/plosone/s/figures

You may also use PLOS’s free figure tool, NAAS, to help you prepare publication quality figures: https://journals.plos.org/plosone/s/figures#loc-tools-for-figure-preparation.

NAAS will assess whether your figures meet our technical requirements by comparing each figure against our figure specifications.

# Author comment: Author response to Decision Letter 2

**DOI:** 10.1371/journal.pone.0357147.r004

2 Jul 2026

Response to reviewers’ comments:

We thank the Reviewers for their valuable comments and suggestions, and specific recommendations. We have addressed all comments and recommendations and hope that the revisions have improved the clarity and strengthened the paper. Below are point-by-point responses to the comments:

Reviewer Comments – PONE-D-26-05748

Reviewer 1

Overall Summary

This manuscript examines body mass index (BMI) and waist circumference (WC) trajectories among midlife women in the SWAN cohort and evaluates their association with incident diabetes. Using group-based trajectory modelling across the first five visits and modified Poisson regression to estimate incidence rate ratios (IRRs) during visits 6–10, the authors identify four trajectory groups for BMI and WC separately, and four joint multi-trajectories. Higher and increasing obesity trajectories were strongly associated with elevated diabetes risk.

The study addresses an important public health question using a well-established longitudinal cohort. The use of trajectory modelling to capture dynamic changes in adiposity is appropriate and potentially informative. However, several methodological and reporting issues require clarification and strengthening before the manuscript can be considered for publication.

Major Comments

1. Ethics Statement – Inconsistent and Potentially Incorrect

The manuscript states:

“This study used publicly available de-identified secondary data… ethical approval was not needed.”

However:

• SWAN is a human subjects study.

• Even for secondary data analyses, authors must state whether their institution deemed the study exempt or not human subjects research.

The current ethics statement is insufficient and potentially non-compliant with journal policy.

Recommendation:

Clarify:

• Whether institutional IRB exemption was obtained.

• Permission to use SWAN study data for manuscript publication

Response:

We conducted this study using de-identified publicly available secondary data from the Study of Women’s Health Across the Nation (SWAN) study accessed through the Inter-university Consortium for Political and Social Research (ICPSR). While the original SWAN study is a human subjects study, our study using publicly available de-identified data is not deemed human subjects research and does not require Downstate IRB review and approval. The original study was approved by the Institutional Review Boards at each participating site, and informed consent was obtained from all participants. We have clarified this in our ethics statement in the Methods section of the revised manuscript: “This study used publicly available de-identified secondary data from the SWAN study accessed through the Inter-university Consortium for Political and Social Research (ICPSR). The original study was approved by the Institutional Review Boards at each participating site, and informed consent was obtained from all participants. Because this study used of publicly available de-identified data, it is deemed not to constitute human subjects research. As such, IRB review and approval was not needed.” (page 4, lines 80-85 in the revised marked manuscript)

It is permissible to publish manuscripts using the publicly available SWAN study data archived at ICPSR. No formal permission is required for individual studies. It has been stated that: “The SWAN Public Use Datasets provide access to longitudinal data describing the physical, biological, psychological, and social changes that occur during the menopausal transition. Data collected from 3,302 SWAN participants from Baseline through Visit 10 are currently available to the public. Users are able to download datasets in a variety of formats, search variables and view recent publications.” (https://www.swanstudy.org/swan-research/data-access/#:~:text=SWAN%20researchers%20have%20published%20over,been%20addressed%20by%20SWAN%20Investigators.)

We have cited individual SWAN study datasets in the methods (references #17-28) in the revised manuscript (page 4, line 79 in the revised marked manuscript).

2. Incident Diabetes Definition – Risk of Misclassification

Incident diabetes was defined via self-report only. No fasting glucose, HbA1c, or medication verification was used.

• The strong IRRs (up to 12.47)

• Only 90 cases

There is risk of misclassification bias, under-diagnosis and differential reporting

Recommendation:

Authors should discuss in this limitation

• Discuss potential misclassification more explicitly.

Response:

Thank you for this recommendation. We have explicitly acknowledged this limitation in the limitations of the revised manuscript: “Incident diabetes was ascertained from self-report alone without clinical or laboratory confirmation. Only 90 cases were identified during sixth to tenth visits. As such there remains the potential for biases from misclassification, under-diagnosis, and differential reporting.” (page 25, lines 408-411 in the revised marked manuscript)

3. Extremely High IRRs – Concern for Overestimation

The reported adjusted IRRs are unusually large:

• BMI very high increasing: IRR 12.47 (5.80–26.78)

• WC very high increasing: IRR 9.79

• Multi-trajectory group 4: IRR 10.43

Given:

• Only 90 total diabetes cases

• Small size of very high groups (~8–10%)

There may be sparse data bias.

Recommendation:

• Provide number of events per group clearly in a table.

• Consider presenting absolute risks.

• Time-to-event modelling may be more appropriate.

Response:

Thank you for these recommendations. We have added a new table, Table 3 that provides the number of incident diabetes by the trajectory groups and rates per 1000 person-years. We have also conducted time-to-event analysis using Cox proportional hazards regression models in the revised manuscript. We have updated the methods, results and discussion accordingly in the revised manuscript.

4. Time-Varying Covariates Not Considered

All covariates are baseline-only.

Given 10-year follow-up:

• Physical activity

• Smoking

• Income

• Menopausal status

likely changed over time.

This may bias results. Authors should justify baseline-only adjustment.

Response:

It is customary to use the baseline covariates using trajectory groups from group-based trajectory modelling as the exposure. We have used baseline-only covariates for adjustment for examining the association between the trajectory groups identified during the first 5 years (visits 1-5) and incident diabetes ascertained during the next 5 years (visits 6-10). We acknowledge that variables or factors such as physical activity, smoking, income, and menopausal status may have changed during the outcome ascertaining 5-year period (visits 6-10). However, these variables or factors can not affect the trajectory groups that have been identified using the BMI or weight circumference from the past during visits 1-5. As such, they cannot be confounders in the trajectory groups – incident diabetes association. The baseline covariates can affect both the trajectory groups and incident diabetes and are appropriate to adjust for as confounders.

Minor Comments

1. Remove extra spacing and formatting issues throughout the manuscript

Response: We have removed extra spacing and tried to adhere to the formatting guidelines provided by the journal. We have inserted figure captions immediately after the first paragraph in which the figure is cited, and inserted tables immediately after the first paragraph in which they are cited.

2. Abstract:

o WC group labelled “very high stable” in methods but “very high increasing” in results. There is inconsistent use of terms.

Response: Thank you for identifying this inconsistency. The waist circumference group should be labelled as “very high stable” in the abstract as labelled in the results. We have corrected this in the abstract.

3. Abstract (Conclusion):

o Authors overstate causality (“could reduce risk”). It’s an observational study, so please rephrase cautiously.

Response: We have rephrased the concluding sentence in the abstract avoiding causality. The revised statement is “Public health interventions on weight management could help reduce the burden of diabetes in this population.” (page 2, lines 26-27 in the revised marked manuscript)

4. Table 1:

o Hispanic participants = 0. This can be removed as Hispanic might be excluded from your study.

Response: We have removed the “Hispanic” category from Table 1.

5. Typographical errors:

o “it’s ability” → “its ability”

o Double dollar sign in income category

Response: We have corrected “it’s ability” to “its ability”, and removed the double dollar sign in income categories in the tables.

6. References:

o Website references should include access date.

Response: We have added the citation date for the website references according to the Vancouver style.

Strengths of the Study

• Use of established longitudinal cohort (SWAN)

• Trajectory-based approach

• Multi-trajectory modeling (advanced method)

• Clear temporal separation between exposure and outcome

• Large sample size for trajectory modeling

Recommendation to Editor

This is a potentially publishable manuscript addressing an important topic. However, significant methodological clarifications and additional analyses are required, particularly regarding:

• Ethics compliance

• Selection bias

• Absolute risks

Response:

We have addressed these issues in our revised manuscript. Please see our responses above.

Reviewer 2

This study provides a comprehensive longitudinal analysis of BMI and waist circumference trajectories among midlife women in the US, focusing on their distinct associations with incident diabetes. By utilizing a multi-trajectory modeling approach with the SWAN dataset, the authors effectively highlight how the progression of adiposity during the menopausal transition serves as a critical predictor of metabolic risk. The findings underscore the clinical importance of early weight management and targeted public health interventions during this specific life stage to reduce the future burden of diabetes.

While the study provides valuable insights, I have two suggestions to further strengthen the validity of the analysis and its clinical implications.

Comment 1: First, I suggest that the authors provide the correlation between BMI and waist circumference. While it is theoretically possible for an individual to have a high BMI but a low waist circumference, demonstrating a strong correlation would justify the use of the four multi-trajectory groups and confirm that these groups adequately cover the majority of the study population.

Response: We thank the reviewer for this suggestion. We have added the correlation between BMI and waist circumference in the revised manuscript (page 5, lines 121-122 in the Methods, and page 7, lines 173-174 in the Results of the revised marked manuscript).

Comment 2: Second, I suggest that the authors further expand the Discussion section by citing additional literature to highlight the long-term impact of interventions during midlife. While I understand the rationale for focusing on the menopausal transition, one could argue that interventions aimed at preventing BMI increases prior to midlife might yield even greater effects on reducing incident diabetes. By discussing the specific magnitude of the impact that midlife interventions can have on long-term health outcomes, the authors would significantly strengthen the clinical significance and research contribution of this study.

Response: We thank the reviewer for this suggestion. We have now added a paragraph in the Discussion citing literature on the long-term impact of interventions during midlife in the revised manuscript (pages 24-25, lines 376-389 in the revised marked manuscript

Attachment Submitted filename: Response to Reviewer Comments_2026 06 05.docx

# Peer review: Decision Letter 2

**DOI:** 10.1371/journal.pone.0357147.r005

**Authors:** Steve Zimmerman

1 Sep 2026

Dear Dr. Brewster,

Thank you for submitting your manuscript to PLOS One. After careful consideration, we feel that it has merit but does not fully meet PLOS One’s publication criteria as it currently stands. Therefore, we invite you to submit a revised version of the manuscript that addresses the points raised during the review process.

The manuscript has been evaluated by two reviewers, and their comments are available below.

Please submit your revised manuscript by Oct 16 2026 11:59PM. If you will need more time than this to complete your revisions, please reply to this message or contact the journal office at plosone@plos.org. When you're ready to submit your revision, log on to https://www.editorialmanager.com/pone/ and select the 'Submissions Needing Revision' folder to locate your manuscript file.

- A letter that responds to each point raised by the academic editor and reviewer(s). You should upload this letter as a separate file labeled 'Response to Reviewers'.
- A marked-up copy of your manuscript that highlights changes made to the original version. You should upload this as a separate file labeled 'Revised Manuscript with Track Changes'.
- An unmarked version of your revised paper without tracked changes. You should upload this as a separate file labeled 'Manuscript'.

If you would like to make changes to your financial disclosure, please include your updated statement in your cover letter. Guidelines for resubmitting your figure files are available below the reviewer comments at the end of this letter.

If applicable, we recommend that you deposit your laboratory protocols in protocols.io to enhance the reproducibility of your results. Protocols.io assigns your protocol its own identifier (DOI) so that it can be cited independently in the future. For instructions see: https://journals.plos.org/plosone/s/submission-guidelines#loc-laboratory-protocols. Additionally, PLOS One offers an option for publishing peer-reviewed Lab Protocol articles, which describe protocols hosted on protocols.io. Read more information on sharing protocols at https://plos.org/protocols?utm_medium=editorial-email&utm_source=authorletters&utm_campaign=protocols.

As the corresponding author, your ORCID iD is verified in the submission system and will appear in the published article. PLOS supports the use of ORCID, and we encourage all coauthors to register for an ORCID iD and use it as well. Please encourage your coauthors to verify their ORCID iD within the submission system before final acceptance, as unverified ORCID iDs will not appear in the published article. *Only*  the individual author can complete the verification step; PLOS staff *cannot*  verify ORCID iDs on behalf of authors.

We look forward to receiving your revised manuscript.

Kind regards,

Steve Zimmerman, PhD

Senior Editor, PLOS One

Journal Requirements:

If the reviewer comments include a recommendation to cite specific previously published works, please review and evaluate these publications to determine whether they are relevant and should be cited. There is no requirement to cite these works unless the editor has indicated otherwise.

[Note: HTML markup is below. Please do not edit.]

Reviewers' comments:

Reviewer's Responses to Questions

**Comments to the Author**

Reviewer #2: All comments have been addressed

Reviewer #3: (No Response)

**********

2. Is the manuscript technically sound, and do the data support the conclusions?

Reviewer #2: Yes

Reviewer #3: Yes

**********

3. Has the statistical analysis been performed appropriately and rigorously?

Reviewer #2: Yes

Reviewer #3: Yes

**********

4. Have the authors made all data underlying the findings in their manuscript fully available?

The PLOS Data policy

Reviewer #2: Yes

Reviewer #3: Yes

**********

5. Is the manuscript presented in an intelligible fashion and written in standard English?

Reviewer #2: Yes

Reviewer #3: Yes

**********

Reviewer #2: Thank you for your consideration for my commments. I confirmed that the authors respond my suggestions. I have no additional comments.

Reviewer #3: 1. The authors primarily rely on BIC, a minimum group size of 5%, and posterior-probability diagnostics. It would be useful to report the BIC values for the competing models and briefly explain how clinical interpretability and trajectory separation contributed to the final four-group solution. This would make the model-selection process more transparent and reproducible.

2. The relatively small number of diabetes events deserves more attention when interpreting the adjusted estimates. Only 90 incident diabetes cases occurred, while several categorical covariates and four-level trajectory variables are included in the adjusted models. Some estimates, particularly for the smaller trajectory groups, have relatively wide confidence intervals. The authors should acknowledge the possibility of limited precision and potential overfitting, and emphasize the confidence intervals rather than relying primarily on statistical significance.

3. The adjustment strategy could be better justified from a clinical and causal perspective. In particular, menopausal status is included as a baseline covariate, although BMI and WC trajectories are themselves closely related to the menopausal transition. Please provide a brief rationale for the covariates included in the final models and clarify whether the intention was to estimate an association independent of baseline menopausal status or to account for potential confounding.

4. Some of the clinical terminology regarding menopause should be revised for accuracy. The statement that natural menopause “occurs in every woman between 45 and 55 years” is somewhat too categorical. Menopause is clinically defined retrospectively after 12 consecutive months of amenorrhea without another cause, and the age range is not restricted to 45–55 years. Since menopausal status is also an adjustment variable in the analysis, a more precise definition would improve the clinical accuracy of the manuscript.

**********

what does this mean?). If published, this will include your full peer review and any attached files.

If you choose “no”, your identity will remain anonymous but your review may still be made public.

**Do you want your identity to be public for this peer review?**  For information about this choice, including consent withdrawal, please see our Privacy Policy

Reviewer #2: No

Reviewer #3: No

**********

[NOTE: If reviewer comments were submitted as an attachment file, they will be attached to this email and accessible via the submission site. Please log into your account, locate the manuscript record, and check for the action link "View Attachments". If this link does not appear, there are no attachment files.]

To ensure your figures meet our technical requirements, please review our figure guidelines: https://journals.plos.org/plosone/s/figures

You may also use PLOS’s free figure tool, NAAS, to help you prepare publication quality figures: https://journals.plos.org/plosone/s/figures#loc-tools-for-figure-preparation.

NAAS will assess whether your figures meet our technical requirements by comparing each figure against our figure specifications.

# Author comment: Author response to Decision Letter 3

**DOI:** 10.1371/journal.pone.0357147.r006

14 Oct 2026

Response to reviewers’ comments:

We thank the Reviewers for their valuable comments and suggestions. We have addressed all comments and hope that the revisions have further improved the clarity and strengthened the paper. Below are point-by-point responses to the comments:

Reviewer Comments – PONE-D-26-05748R2

Reviewer #2:

Comment: Thank you for your consideration for my comments. I confirmed that the authors respond my suggestions. I have no additional comments.

Response: We thank Reviewer #2.

Reviewer #3:

Comment 1: The authors primarily rely on BIC, a minimum group size of 5%, and posterior-probability diagnostics. It would be useful to report the BIC values for the competing models and briefly explain how clinical interpretability and trajectory separation contributed to the final four-group solution. This would make the model-selection process more transparent and reproducible.

Response: Thank you for this helpful comment. We have clarified the model-selection process in the Methods and added the BIC values for the competing models in Supplementary S1 Table. We have added the following in the Methods: “Models The model with the different numbers of trajectory groups were compared using the lowest Bayesian Information Criterion (BIC) as a measure of goodness-of-fit, where the groups with the requirement that each group included at least 5% of the study participants was selected. BIC values for the competing models are presented in S1 Table. The four-group model was selected based on the combined consideration of BIC, minimum group size, trajectory separation, and clinical interpretability. Compared with the three-group model, the four-group model distinguished two clearly separated intermediate BMI trajectories that were combined into a single trajectory in the three-group model. The four trajectories also yielded four clinically distinguishable BMI trajectories corresponding approximately to healthy weight, overweight, class 1 obesity and class 3 obesity with all groups exceeding the 5% minimum group size. The five-group model included an additional trajectory group consisting of only 2.1% of participants below the prespecified 5% minimum group size.” (lines 125-136 in the revised marked manuscript)

Comment 2: The relatively small number of diabetes events deserves more attention when interpreting the adjusted estimates. Only 90 incident diabetes cases occurred, while several categorical covariates and four-level trajectory variables are included in the adjusted models. Some estimates, particularly for the smaller trajectory groups, have relatively wide confidence intervals. The authors should acknowledge the possibility of limited precision and potential overfitting, and emphasize the confidence intervals rather than relying primarily on statistical significance.

Response: We thank the reviewer for highlighting the relatively small number of incident diabetes events and its implications for the adjusted estimates. We agree that this needs to be acknowledged in the limitations of the study. We have added the following in the limitations of the Discussion: “Only 90 incident diabetes cases were identified during the sixth to tenth visits. The relatively small number of incident diabetes occurring during the follow-up period may have resulted in HR estimates to be imprecise with wide 95% CIs and increased the potential for model overfitting, particularly for covariate categories with few events. Thus, these findings should be interpreted cautiously considering both the magnitude and 95% CIs rather than statistical significance alone.” (lines 377-382 in the revised marked manuscript)

Comment 3: The adjustment strategy could be better justified from a clinical and causal perspective. In particular, menopausal status is included as a baseline covariate, although BMI and WC trajectories are themselves closely related to the menopausal transition. Please provide a brief rationale for the covariates included in the final models and clarify whether the intention was to estimate an association independent of baseline menopausal status or to account for potential confounding.

Response: We thank the reviewer for this comment. We agree that the rationale for the covariate adjustment strategy should be clarified. The covariates included in the final models were selected a priori based on their clinical relevance to both adiposity trajectories and diabetes risk and their potential to account for baseline differences between trajectory groups. Menopausal status was included as a baseline covariate because menopausal status is clinically related to changes in body composition and adiposity as well as to diabetes risk. Our intention was to account for potential differences in diabetes risk associated with baseline menopausal status and thereby estimate the association between BMI and waist circumference trajectory group and incident diabetes conditional on baseline menopausal status. We recognize that the menopausal transition may itself contribute to changes in BMI and waist circumference over time. We have added the following to the Methods: “These covariates were selected a priori based on their clinical relevance to adiposity and diabetes risk, and their potential to account for baseline differences between adiposity trajectory groups. Baseline menopausal status was included to account for differences in adiposity patterns and diabetes risk associated with menopausal status.” (lines 151-155 in the revised marked manuscript)

Comment 4: Some of the clinical terminology regarding menopause should be revised for accuracy. The statement that natural menopause “occurs in every woman between 45 and 55 years” is somewhat too categorical. Menopause is clinically defined retrospectively after 12 consecutive months of amenorrhea without another cause, and the age range is not restricted to 45–55 years. Since menopausal status is also an adjustment variable in the analysis, a more precise definition would improve the clinical accuracy of the manuscript.

Response: Thank you for this helpful comment. We agree that the original statement in the Introduction that natural menopause “occurs in every woman between 45 and 55 years” was overly categorical and did not accurately reflect the clinical definition of menopause. We have revised the statement and added the following: “Menopause is the biological transition that naturally occurs in every woman’s life at the end of reproductive years. Natural menopause is clinically defined retrospectively after 12 consecutive months of and is characterized by the cessation of menstruation due to decreased ovarian function in absence of any other physiological or pathological cause, or clinical intervention [4].” (lines 33-36 in the revised marked manuscript). We have also added the definition of premenopausal and early perimenopausal status in the Methods in lines 83-85 in the revised marked manuscript.

Attachment Submitted filename: Response to Reviewer Comments_2026 09 13.docx

# Peer review: Decision Letter 3

**DOI:** 10.1371/journal.pone.0357147.r007

**Authors:** Jihan Kim

1 Nov 2026

Body mass index and waist circumference trajectories among midlife women in the United States and their association with incident diabetes: The Study of Women’s Health Across the Nation

PONE-D-26-05748R3

Dear Dr. Brewster,

We’re pleased to inform you that your manuscript has been judged scientifically suitable for publication and will be formally accepted for publication once it meets all outstanding technical requirements.

Within one week, you’ll receive an e-mail detailing the required amendments. When these have been addressed, you’ll receive a formal acceptance letter and your manuscript will be scheduled for publication.

An invoice will be generated when your article is formally accepted. Please note, if your institution has a publishing partnership with PLOS and your article meets the relevant criteria, all or part of your publication costs will be covered. Please make sure your user information is up-to-date by logging into Editorial Manager at Editorial Manager® and clicking the ‘Update My Information' link at the top of the page. For questions related to billing, please contact billing support.

If your institution or institutions have a press office, please notify them about your upcoming paper to help maximize its impact. If they’ll be preparing press materials, please inform our press team as soon as possible -- no later than 48 hours after receiving the formal acceptance. Your manuscript will remain under strict press embargo until 2 pm Eastern Time on the date of publication. For more information, please contact onepress@plos.org.

Kind regards,

Jihan Kim

Academic Editor

PLOS One

Additional Editor Comments (optional):

Reviewers' comments:

Reviewer's Responses to Questions

**Comments to the Author**

Reviewer #2: All comments have been addressed

Reviewer #3: All comments have been addressed

**********

2. Is the manuscript technically sound, and do the data support the conclusions?

Reviewer #2: Yes

Reviewer #3: Yes

**********

3. Has the statistical analysis been performed appropriately and rigorously?

Reviewer #2: Yes

Reviewer #3: Yes

**********

4. Have the authors made all data underlying the findings in their manuscript fully available?

The PLOS Data policy

Reviewer #2: Yes

Reviewer #3: Yes

**********

5. Is the manuscript presented in an intelligible fashion and written in standard English?

Reviewer #2: Yes

Reviewer #3: Yes

**********

Reviewer #2: Suggested comment to the authors

Minor comments / Typographical and grammatical corrections

The manuscript has been substantially improved following revision. However, I noticed several typographical and grammatical errors that should be corrected before publication. Please carefully proofread the manuscript, including the following points:

In the Abstract (Results),“with a higher rates of diabetes” should be corrected to“with higher rates of diabetes.”

In the Background,“diabetes was directly accounted for 1.6 million deaths” should be corrected to“diabetes directly accounted for 1.6 million deaths.”

In the Background,“the risk of type diabetes” should presumably be“the risk of type 2 diabetes.”

In the Methods,“Weight circumference (cm) was measured at each visit” should be corrected to“Waist circumference (cm) was measured at each visit.”

In the Results,“$$50,000 to $99,999” should be corrected to“$50,000 to $99,999.” This error appears more than once in the manuscript.

In the waist circumference trajectory section, the sentence“The third trajectory group (high increasing) comprised of 18.7% women had a waist circumference...” is grammatically incorrect and should be revised. For example:“The third trajectory group (high increasing) comprised 18.7% of women who had a waist circumference...”

Overall, these appear to be minor editorial issues and do not affect the main conclusions of the study, but careful proofreading would improve the clarity and presentation of the manuscript.

Reviewer #3: The authors have adequately addressed the reviewers’ comments and improved the clarity and transparency of the manuscript, particularly regarding trajectory model selection and the interpretation of the findings in light of the limited number of incident diabetes cases. The revisions are satisfactory, and I recommend acceptance of the manuscript for publication.

**********

what does this mean?). If published, this will include your full peer review and any attached files.

If you choose “no”, your identity will remain anonymous but your review may still be made public.

**Do you want your identity to be public for this peer review?**  For information about this choice, including consent withdrawal, please see our Privacy Policy

Reviewer #2: No

Reviewer #3: No

**********

# Editor report: Acceptance letter

**DOI:** 10.1371/journal.pone.0357147.r008

**Authors:** Jihan Kim

PONE-D-26-05748R3

PLOS One

Dear Dr. Brewster,

I'm pleased to inform you that your manuscript has been deemed suitable for publication in PLOS One. Congratulations! Your manuscript is now being handed over to our production team.

At this stage, our production department will prepare your paper for publication. This includes ensuring the following:

* All references, tables, and figures are properly cited

* All relevant supporting information is included in the manuscript submission,

* There are no issues that prevent the paper from being properly typeset

You will receive further instructions from the production team, including instructions on how to review your proof when it is ready. Please keep in mind that we are working through a large volume of accepted articles, so please give us a few days to review your paper and let you know the next and final steps.

Lastly, if your institution or institutions have a press office, please let them know about your upcoming paper now to help maximize its impact. If they'll be preparing press materials, please inform our press team within the next 48 hours. Your manuscript will remain under strict press embargo until 2 pm Eastern Time on the date of publication. For more information, please contact onepress@plos.org.

You will receive an invoice from PLOS for your publication fee after your manuscript has reached the completed accept phase. If you receive an email requesting payment before acceptance or for any other service, this may be a phishing scheme. Learn how to identify phishing emails and protect your accounts at https://explore.plos.org/phishing.

If we can help with anything else, please email us at customercare@plos.org.

Thank you for submitting your work to PLOS One and supporting open access.

Kind regards,

PLOS One Editorial Office Staff

on behalf of

Dr. Jihan Kim

Academic Editor

PLOS One
